# Apolipoprotein A1 deficiency increases macrophage apoptosis and necrotic core development in atherosclerotic plaques in a Bim-dependent manner

**DOI:** 10.1016/j.jlr.2025.100782

**Published:** 2025-03-20

**Authors:** Alexander S. Qian, George E.G. Kluck, Pei Yu, Leticia Gonzalez, Elizabeth Balint, Bernardo L. Trigatti

**Affiliations:** Thrombosis and Atherosclerosis Research Institute, Centre for Metabolism, Obesity and Diabetes Research, and Department of Biochemistry and Biomedical Sciences, McMaster University and, Hamilton Health Sciences, Hamilton, Ontario, Canada

**Keywords:** apolipoprotein A1, apoptosis, atherosclerosis, HDL, macrophages, monocytes

## Abstract

In advanced atherosclerotic lesions, macrophage apoptosis contributes to plaque progression and the formation of necrotic cores, rendering plaques vulnerable to rupture. The proapoptotic protein B-cell lymphoma 2 [Bcl-2] interacting mediator of cell death (Bim) plays a crucial role in mediating apoptosis in macrophages under prolonged endoplasmic reticulum stress. HDL has been shown to suppress macrophage apoptosis induced by endoplasmic reticulum stressors. To investigate the impact of apolipoprotein A1 (ApoA1) deficiency, associated with reduced HDL levels, on necrotic core growth and plaque apoptosis, we introduced *ApoA1* deficiency into low-density lipoprotein receptor (LDLR) knockout mice and fed them a high-fat diet for 10 weeks. ApoA1-deficient *Ldlr* KO mice developed advanced plaques characterized by large necrotic cores, increased apoptosis, and elevated *Bim* expression in macrophages within the plaques. To assess whether deletion of *Bim* could mitigate this development, mice underwent bone marrow transplantation with bone marrow from either Bim-deficient mice or from mice with a deletion of myeloid-derived Bim driven by *LyzM-cre*. Inhibiting Bim in all bone marrow-derived cells led to leukocytosis, reductions in plasma cholesterol and triglyceride levels, and decreased plaque apoptosis, necrotic core, and plaque sizes in *ApoA1* and *Ldlr* double-KO mice but not in *Ldlr* KO mice. Likewise, conditional deletion of Bim in the myeloid compartment of *ApoA1* and *Ldlr* double-KO mice also reduced apoptosis, necrotic core sizes, and plaque sizes, without inducing leukocytosis or lowering plasma cholesterol levels. These findings suggest that ApoA1 deficiency triggers apoptosis in myeloid cells through a Bim-dependent pathway, significantly contributing to the development of necrotic cores and the progression of atherosclerotic plaques.

Atherosclerosis is a complex disease that results in the formation of lipid-rich plaques within the walls of arteries and is the underlying cause of several cardiovascular diseases (1, 2). Atherosclerosis begins in the regions of disturbed blood flow, triggering endothelial dysfunction and monocyte recruitment (3). These monocytes differentiate into macrophages to facilitate the uptake of modified lipoproteins including oxidized low-density lipoproteins (oxLDL) forming lipid-laden foam cells that form the basis of the atherosclerotic lesion (2, 4, 5). As plaques advance, they develop large necrotic cores, characterized by necrotic cellular debris, inflammatory mediators, matrix-degrading proteases, tissue factor, and extracellular lipids (6). Large necrotic cores and thinning of the fibrous cap increase the risk of plaque rupture and symptomatic cardiovascular events such as myocardial infarction (7, 8, 9).

Cell death of macrophages and foam cells within plaques is a crucial driver of necrotic core development in atherosclerosis (9, 10). Macrophage cell death in atherosclerotic plaques can occur through various pathways including apoptosis, and regulated and secondary necrosis pathways (reviewed in Puylaert *et al.* (11)). Necroptosis is one regulated necrosis pathway shown to occur in atherosclerotic plaques which can be activated in macrophages in response to oxLDL or exposure to tumor necrosis factor alpha under conditions when apoptosis is blocked (12, 13). Necroptosis leads to cell membrane rupture and the release of cell debris which acts as highly inflammatory damage-associated molecular patterns (14). In contrast, apoptotic cell death is typically noninflammatory, as apoptotic cells are efficiently removed by efferocytosis (15, 16, 17). Inhibiting macrophage apoptosis early in lesion development has been shown to accelerate plaque progression in several mouse studies (17, 18, 19, 20, 21). However, as atherosclerotic plaques develop, efferocytosis is impaired, leading to the accumulation of apoptotic cells and increasing apoptotic bodies susceptible to secondary necrosis (15, 16, 22, 23, 24, 25). As a result, apoptotic cell death in the plaque is a meaningful contributor to necrotic core expansion as plaques advance (17, 23, 25).

In plaques, accumulation of unesterified cholesterol and other triggers of prolonged endoplasmic reticulum (ER) stress in macrophages activate apoptosis through the intrinsic mitochondrial pathway of apoptosis (26, 27, 28). Markers of ER stress have been reported at all stages of atherosclerotic plaque development and alleviation of ER stress has been reported to protect against atherosclerosis (29, 30, 31). A key mediator of macrophage apoptosis in response to ER stress is Bim (B-cell lymphoma 2 [Bcl-2] interacting mediator of cell death), a member of the Bcl-2 family of apoptotic regulators (32, 33). Prolonged ER stress can upregulate *Bim* expression through various branches of the unfolded protein response (34). Specifically, the pathway involving CCAAT box–binding enhancer protein homologous protein (CHOP, also known as GADD153) is essential for ER stress-induced apoptosis by upregulating *Bim* transcription in macrophages and controlling necrotic core growth and apoptosis in atherosclerotic mice (34, 35). Furthermore, ER stress can activate protein phosphatase 2A (PP2A), which dephosphorylates Bim, preventing its ubiquitination and proteasomal degradation (34). Additionally, C-Jun N-terminal kinase activation by the inositol-requiring transmembrane kinase/endoribonuclease 1α (IRE1α) branch of the unfolded protein response can also post translationally activate longer isoforms of Bim by phosphorylation (36, 37, 38). Cytosolic calcium influx, occurring during ER stress, induces Bim's inhibitory interactions with antiapoptotic protein, B cell lymphoma-2 (Bcl-2) (39). Therefore, prolonged ER stress increases Bim expression and activity and reduces its degradation, thereby promoting Bim-mediated induction of apoptosis.

Bim is an intrinsically unstructured protein with a Bcl-2 homology 3 (BH3) domain (33). This domain enables Bim to inhibit antiapoptotic Bcl-2 family members, such as Bcl-2, preventing their interaction with and inhibition of proapoptotic members like Bax and Bak (40). Consequently, Bax and Bak assemble, forming pores in the mitochondrial membrane, facilitating cytochrome C release, and apoptosome formation (40). Bim can also interact with these proapoptotic members, promoting their mitochondrial association (41, 42, 43). Ubiquitously expressed in all tissues, Bim plays a crucial role in controlling autoimmunity in lymphocytes (32). Bim-deficient mice exhibit increased concentrations of circulating leukocytes, which are more resistant to apoptosis (44). Notably, Bim deficiency in macrophages can reduce ER stress-induced apoptosis (34) making it a potential target worth investigating.

We have previously reported that HDL protects cultured macrophages from both apoptotic and necroptotic cell death (13, 45, 46). HDL-mediated protection of macrophages against apoptosis and necroptosis both appear to be mediated through activation of the phosphoinositide 3-kinase (PI3K)/protein kinase B (Akt) cell survival pathway (13, 45, 46). Apolipoprotein A-1 (ApoA1) is the major apolipoprotein component of high-density lipoprotein (HDL). Genetic deficiency of ApoA1 in atherosclerotic low-density lipoprotein receptor (*Ldlr*) and apolipoprotein E (*ApoE*) KO mice leads to reduced HDL (13, 47, 48, 49). These atherosclerotic mice also develop plaques with larger necrotic cores (13, 47, 48, 49). Conversely, overexpression of ApoA1 reduces atherosclerotic plaque and necrotic core development in transgenic mice (50, 51, 52, 53). These studies provide strong evidence for the atheroprotective effects of ApoA1. However, the effect of ApoA1 deficiency on macrophage apoptosis during atherosclerosis development in vivo has not been fully explored.

In this study, we demonstrate that inactivation of *ApoA1* expression in *Ldlr*^KO/KO^ mice increases atherosclerotic plaque and necrotic core size as well as Bim protein levels and apoptosis within atherosclerotic plaques. We demonstrate that the inactivation of Bim expression in all bone marrow (BM) derived cells results in leukocytosis, reduction in plasma cholesterol and triglyceride levels, and protections against the increased atherosclerosis plaque development of *ApoA1*^KO/KO^*Ldlr*^KO/KO^ mice. Therefore, we demonstrate that the protection against increased atherosclerosis, necrotic core development, and apoptosis is the result of Bim deficiency in BM-derived myeloid cells, whereas the increased leukocytosis and reduced plasma cholesterol and triglyceride levels reflect Bim deficiency in nonmyeloid cells derived from the BM. These findings illustrate that macrophage Bim plays a key role in the enhanced apoptosis within atherosclerotic plaques resulting from ApoA1 deficiency, suggesting it may be a potential target for future interventions.

## Materials and Methods

### Mice

Animal care and experiments on mice followed procedures approved by the McMaster University Animal Research Ethics Board and in accordance with Canadian Council on Animal Care guidelines. Parental *Ldlr*^KO/KO^ (B6.129S7-*Ldlr*^*t*m1Her^/J Stain #002207), *ApoA1*^KO/KO^ (B6.129P2-*Apoa1*^tm1Unc^/J Stain #002055), and *APOA1*^TG/TG^ (C57BL/6-Tg(*APOA1*)1Rub/J) mice were purchased from Jackson Laboratory (Bar Harbor, ME). *Ldlr*^KO/KO^ crossed with either *ApoA1*^KO/KO^ or *APOA1*^TG/TG^ mice were generated by mating through two or three generations selecting for the double homozygous mutants. Wild-type (*WT*) C56BL/6J (Strain #000664) mice *Bim*^KO/KO^ (B6.129S1-*Bcl2l11*^tm1.1Ast^/J Strain #004525) mice were obtained from the Jackson Laboratory. Mice with the *Bim* gene flanked with loxP sites (*Bim*^fl/fl^) were graciously donated by and previously described by Dr David Hildeman at the Cincinnati Children's Hospital Medical Center (Cincinnati, OH) (54, 55). Mice with conditional myeloid deletions of *Bim* (*LyzM*^cre/cre^*Bim*^fl/fl^) were generated by mating *Bim*^fl/fl^ with *LyzM*^cre/cre^ (B6.129P2-*Lyz2*^*tm1(cre)Ifo*^/J Stain #004781) mice originally obtained from Jackson Laboratories. All mice were maintained as breeding colonies in a Helicobacter-positive environment in the David Braley Research Institute Animal Facility at McMaster University. Mice were housed in high efficiency particulate arresting-filtered ventilated cages with automatic watering and had free access to a standard laboratory diet (Teklad 18% protein diet, CAT#2918-032222M; Harlan Laboratories, Mississauga, ON, Canada). Genotyping was carried out by PCR of tail biopsy-derived DNA.

### Aortic sinus atherosclerosis model

Atherosclerosis was induced by feeding a high-fat diet (HFD) (#112286, Dyets Inc, Bethlehem, PA) with 21% (wt/wt) butterfat, 0.15% (wt/wt) cholesterol, and 1% safflower oil for 10 weeks, beginning at 10 weeks of age. At the end of the HFD feeding period, mice were fasted overnight, weighed, and sacrificed for subsequent analysis. Hearts were freshly embedded and frozen in Epredia Cryomatrix embedding medium (#6769006, Thermo Fisher Scientific, Ottawa, ON, Canada), then stored at −80°C. Subsequently, serial cross sections (covering up to 600 μm of the aortic sinus from the base of the aortic annulus) of 10 μm thickness were sectioned on a cryostat and mounted onto Superfrost microscope slides (#4951PLUS4600621, Thermo Fisher Scientific). For atherosclerotic plaque size determination, frozen sections were treated with 37% formaldehyde for one minute, rinsed in water, and stained with Oil Red O for 10 minutes and separately with Mayer's hematoxylin and eosin (H&E) for atherosclerotic plaque size and necrotic core analysis. In bone marrow transplantation (BMT) experiments, hearts were fixed in 10% formalin, processed, and embedded in paraffin wax, and sectioned in 5 μm thick serial cross-sections (covering up to 600 μm of the aortic sinus from the base of the aortic annulus). For atherosclerosis plaque size and necrotic core size analysis, sections were deparaffinized in a procedure described previously (56) and stained with Mayer's H&E. Images were captured using the Zeiss Axiovert 200 M inverted microscope (Carl Zeiss Canada Ltd., Toronto, ON, Canada). Atherosclerotic plaque profiles and necrotic core areas (anuclear areas negative for staining) were analyzed using AxioVision v3.1.2.1 software (Carl Zeiss Canada Ltd.). The plaque area represents the amount of plaque area at the peak of each plaque profile. The plaque volumes were calculated as the area under the curve from each plaque profile in GraphPad Prism (v10.4.1, GraphPad, Boston, MA). To minimize observer error and bias, images were analyzed in a blinded manner by two individuals.

### Bone marrow transplantation

Prior to BMT, BM cells were harvested from either male WT, *Bim*^KO/KO^, *LyzM*^cre/cre^, or *LyzM*^cre/cre^*Bim*^fl/fl^ mice up to six months of age. BM cells were resuspended in Iscove's Modified Dulbecco's medium (31980030, Thermo Fisher Scientific) containing 2% heat-inactivated fetal bovine serum (FBS), 2 mM L-glutamine, and 50 U/ml penicillin and streptomycin as previously described (13, 45, 57). Male *Ldlr*^KO/KO^ and *ApoA1*^KO/KO^*Ldlr*^KO/KO^ BM recipients (10–12 weeks of age) were exposed to a total body dose of 14 Gy of Cs^13^^7^ irradiation, delivered as a split (2/3,1/3) dose with a 3-h rest between. Immediately following irradiation, BM cells (3 × 10^6^ cells) were injected retroorbitally. Transplanted mice were tagged and coded and allowed to recover for 2 weeks in new cages with sterile food, mush, water, and hydrating gel on placed heating pads. After recovery, mice were placed in new cages on ventilated racks with unlimited access to standard diet and water. Four weeks following BMT, mice were fed an HFD for 10 weeks unless otherwise indicated. At the end of HFD feeding period, mice were fasted overnight, weighed, and sacrificed as described above. Successful BMTs were verified by PCR of blood DNA.

### Plasma analysis

Plasma lipid measurements were obtained from plasma samples using commercially available enzymatic color assays. Plasma total cholesterol, free cholesterol, HDL cholesterol, and triglycerides were quantified using the following assay kits: Cholesterol Infinity (TR13421; Thermo Fisher Scientific), Free Cholesterol E (993-02501; Wako Diagnostics, Mountain View, CA), HDL Cholesterol E (997-01301; Wako Diagnostics), and L-Type Triglyceride M (998-02992; Wako Diagnostics). Measurements were conducted following the respective manufacturers' instructions. Triglyceride secretion was measured in plasma samples from fasted *Ldlr*^KO/KO^ BM recipients (as described above) treated with the lipoprotein lipase inhibitor 500 mg/kg tyloxapol intravenously through retroorbital injection as described in a prior studies (58, 59). Blood was collected prior to and hourly following lipoprotein lipase inhibition and triglyceride concentration in plasma was measured as described above. Plasma interleukin-6 (IL-6) concentrations were quantified using an ELISA kit for mouse IL-6 (431304; BioLegend, San Diego, CA) following the manufacturer's instructions.

### Flow cytometry

To analyze blood leukocyte (CD45+) concentrations of B cells (B220+), T cells (CD3+), monocytes (CD11b + Ly6G-), and corresponding cell subsets by flow cytometry, two portions of the blood collected was incubated with fluorescently labeled antibodies. Red blood cells were lysed and samples were fixed with 1-step Fix/Lyse Solution (00-5333-54, Thermo Fisher Scientific) for 15 min. After washing, all samples were incubated in the dark for 30 min with Brilliant Violet 510 Anti-Mouse CD45 (103138, BioLegend; 1:100), PerCP-Cyanine5.5 Anti-Human/Mouse CD45R (B220) antibody (45-0452-80, Thermo Fisher Scientific; 1:100), FITC Rat Anti-Mouse CD3 antibody (555274, BD Biosciences, Mississauga, ON, Canada; 1:100) and unconjugated Anti-Mouse CD16/CD32 (101302, BioLegend; 1:50). One set of samples were also incubated with PE Anti-Mouse NK1.1 (553165, BD Biosciences; 1:50), PE Cy5 Anti-Mouse CD4 (553050, BD Biosciences; 1:50), and PE Cy7 Anti-Mouse CD8a (552877, BD Biosciences; 1:50). A duplicate set of samples were instead incubated with PE Anti-Mouse CD11b antibody (M1/70.15) (RM2805, Thermo Fisher Scientific; 1:50), PE Cy7 Anti-Mouse Ly6C antibody (128018, BioLegend; 1:50), and PE Dazzle-594 Anti-Mouse Ly6G (127648, BioLegend; 1:50). Samples were run on a BD FACSMelody system (BD Biosciences) and analyzed using FlowJo (v10.8.1, BD, Ashland, OR). Gating for lymphocytes is outlined in supplemental Fig. S1 and gating for myeloid cells is outlined in supplemental Fig. S2. Absolute concentrations were determined using a formula accounting for obtained counts of 123Count eBeads (Thermo Fisher Scientific) from each sample.

### Immunofluorescence staining in plaques

For immunofluorescence staining of aortic sinus atherosclerosis from *Ldlr*^KO/KO^ and *ApoA1*^KO/KO^*Ldlr*^KO/KO^ mice, frozen sections were fixed in 1% paraformaldehyde (PFA) for 5 min at room temperature before being immersed in a 2:1 ethanol:acetic acid solution for five minutes at −20°C. For immunofluorescence staining of aortic sinus atherosclerosis from BMT mice, paraffin sections were deparaffinized as described previously (56). Antigen retrieval of aortic sinus sections was conducted using immersion in citric acid-based antigen unmasking solution (#H-3300, Vector Labs, Newark, CA) for 10 min in a preheated pressure cooker. Slides were washed with PBS and incubated with PBS containing 0.1% Triton X-100 for 5 min at room temperature. Afterward, all sections were washed and blocked using PBS containing 5% goat serum for 1 h at room temperature. Sections of the peak plaque area were selected and incubated with antibodies overnight at 4°C: rabbit cleaved caspase 3 (CC3) (#9661, Cell Signaling Technology Inc.; 1:200), rat Mac-3 (CD107b; 553322, BD Biosciences; 1:50), rabbit Bim (C34C5; #2933, Cell Signaling Technology Inc.; 1:200), rabbit CHOP/GADD153 (F-168; sc-575, Santa Cruz Biotech., Dallas, TX; 1:40), rabbit phosphorylated mixed lineage kinase domain like pseudokinase (pMLKL; Ser 345; D6E3G; 37333, Cell Signaling Technologies Inc.; 1:200), rabbit IL-6 (ab6672, Abcam, Waltham, MA; 1:100), or mouse Alexa Fluor 594 conjugated CD45.2 (109850, Biolegend; 1:100). Following incubation, slides were washed with PBS with 0.5% Tween-20 three times for 10 min and corresponding goat secondary antibody conjugated to Alexa Fluor 488 or Alexa Fluor 594 (Thermo Fisher Scientific; 1:500) was added for 1 h at room temperature. All sections were counterstained with 4',6-diamidino-2-phenylindole (DAPI) to visualize cell nuclei and mounted with PermaFluor mounting medium (TA030FM, Thermo Fisher Scientific). For GRP78 immunofluorescence staining in plaques, mouse KDEL (10C3; ADI-SPA-827-D, Enzo Life Sciences, Farmingdale, NY; 1:100) antibody was used in conjunction with the Mouse-on-Mouse Detection Kit (BMK-2202, Vector Labs) following manufacturer's procedures. Rabbit mAb IgG isotype control (DA1E; 3900S, Cell Signaling Technology; 1:100) or rat mAb IgG isotype control (400402, Biolegend; 1:50) was used as a negative control (supplemental Fig. S3). DNA fragmentation in aortic sinus atherosclerosis sections was fluorescently stained green with FITC by TUNEL using the ApopTag Fluorescein In Situ Apoptosis Detection kit (S7110, EMD Millipore, Burlington, MA) following manufacturer's instructions. All sections were counterstained with 300 nM of DAPI for 5 min at room temperature to visualize cell nuclei and then mounted with PermaFluor mounting medium (TA030FM, Thermo Fisher Scientific). Images of stained immunofluorescent sections were captured using the Leica SP5 Confocal Microscope (Leica Camera, London, ON, Canada). Quantification of positive CC3, Mac-3, pMLKL, IL-6, and CD45 staining was taken as a threshold area relative to plaque area. Quantification of Bim staining was taken as Bim positive area over Mac-3 positive area within the plaque. Cell nuclei positive for TUNEL staining were taken as a percentage of total DAPI-positive nuclei within the plaque. All immunofluorescent images were quantified and analyzed using ImageJ software.

### Isolation and culture of mouse peritoneal macrophages

Mice were injected intraperitoneally with 1 ml of 10% thioglycolate broth medium (T9032, Sigma-Aldrich, Oakville, ON, Canada) to elicit macrophages to the peritoneum. After four days, mice were euthanized, and murine peritoneal macrophages were harvested by peritoneal lavage using a PBS at pH 7.4 solution containing 5 mM of EDTA. Cells were cultured on 8-well chamber slides at 1.5 × 10^5^ cells per well in Dulbecco's modified Eagle's medium (DMEM) containing 10% FBS, 2 mM L-glutamine and 50 U/ml penicillin and streptomycin. Following two hours of allowing macrophages to adhere to slides, DMEM with 10% FBS is replaced with DMEM with 3% newborn calf lipoprotein deficient serum as previously described (60) and cultured for 1 day. Cells were incubated for 24 h with the absence or presence of 50 μg/ml of human HDL (J64903, Alfa Aesar, Haverhill, MA or 12-16-080412, Athens Research and Technology, Athens, GA) or 50 μg/ml of human ApoA1 (16-6-120101, Athens Research and Technology) along with differing apoptotic inducers including oxLDL (100 μg/ml, J65591, Alfa Aesar), 7-ketocholesterol (10 μg/ml; Cayman Chemical, Ann Arbor, MI), or tunicamycin (TN; 10 μg/ml; Cayman Chemical). For necroptosis induction, macrophages were treated with or without 50 μg/ml oxLDL (J65591, Alfa Aesar) with the presence of 50 μM carbobenzoxy-valyl-alanyl-aspartyl-[O-methyl]-fluoromethylketone (ZVAD.fmk) (A1902, APExBIO, Houston, TX) and with or without 50 μM necrostatin-1s (Nec1s; #2535-1, Biovision, Paris, France) for 24 h in DMEM with 3% newborn calf lipoprotein deficient serum. Cholesterol efflux from macrophages to human HDL or human ApoA1 was measured using a cell-based cholesterol efflux assay kit (ab196985, Abcam) following manufacturer's instructions.

### Immunocytochemistry

For fluorescent staining in apoptotic macrophages, cells were fixed in 2% PFA for 20 min after corresponding treatments. Fluorescent staining for DNA fragmentation by TUNEL using the ApopTag Fluorescein In Situ Apoptosis Detection kit (S7110, EMD Millipore) and counterstained with DAPI. CC3 was detected by immunofluorescence using CC3 (Asp175) antibody (#9661, Cell Signaling Technology Inc.; 1:200). Immunofluorescence staining for Bim in cells from WT and *Bim*^KO/KO^ macrophages was probed with a Bim (C34C5) antibody (#2933, Cell Signaling Technology Inc.; 1:200). Alexa Fluor 488 goat anti-rabbit IgG (A-11008, Thermo Fisher Scientific; 1:500) was used as a secondary antibody. Staining for necroptotic macrophages required incubation with 1 μg/ml of propidium iodide (Sigma-Aldrich) for 10 min on ice before fixation with 2% PFA for 20 min. All cells were counterstained with DAPI for 5 min at room temperature to visualize cell nuclei and mounted with PermaFluor mounting medium (TA030FM, Thermo Fisher Scientific). Images were captured using the Zeiss Axiovert inverted microscope with a minimum of 4 fields of view per treatment and quantified as a percentage of total DAPI-positive nuclei using ImageJ software.

### Western blots

Cell lysates were prepared from murine peritoneal macrophage as described above with changes to experimental conditions depending on the experiment indicated in the results sections. Cell lysates were generated on ice employing RIPA buffer (50 mM Tris-HCl pH 7.4; 150 mM NaCl; 1% Triton X-100; 1% sodium deoxycholate; 0.1% SDS; and 1 mM EDTA) combined with protease inhibitors (1 mg/ml pepstatin A, #77170, Sigma-Aldrich; 1 mg/ml leupeptin, #L2884, Sigma-Aldrich; 10 mg/ml aprotinin, #A1153, Sigma-Aldrich; and 50 mM phenylmethanesulfonyl fluoride, #10837091001, Sigma-Aldrich) and a 1X PhosSTOP phosphatase inhibitor cocktail (#04906845001, Roche, Mississauga, ON, Canada). Samples comprising 30 μg of protein, as determined by a BCA assay, were subjected to SDS-PAGE, and the proteins were subsequently transferred onto PVDF membranes for immunoblotting. These membranes were blocked with tris-buffered saline supplemented with 0.1% tween-20 and 5% BSA for one hour at room temperature. Membranes were incubated overnight at 4 °C with primary antibodies against Bim (C34C5) (#2933, Cell Signaling Technology Inc, Danvers MA; 1:1000), GADD153/CHOP (F-168; sc-575, Santa Cruz Biotech.; 1:200), and KDEL (10C3; ADI-SPA-827, Enzo Life Sciences; 1:1000). Antibody against β-actin (13E5; Peroxidase-conjugated; #5124, Cell Signaling Technology Inc; 1:5000) was used as a loading control. Secondary antibodies peroxidase-conjugated AffiniPure Donkey Anti-Rabbit IgG (711-035-152, Jackson ImmunoResearch Labs., West Grove, PA; 1:5000) or peroxidase-conjugated AffiniPure Donkey Anti-Mouse IgG (715-035-150, Jackson ImmunoResearch Labs.; 1:5000) were applied after primary antibody incubation following three 10-min washes in tris-buffered saline supplemented with 0.1% tween-20 for 1 h at room temperature. The visualization of proteins was carried out by enhanced chemiluminescence (ECL; Pierce ECL Western Blotting Substrate, Thermo Fisher Scientific) and captured with a ChemiDoc XRS imaging system (Bio-Rad, Hercules, CA).

### Statistical analysis

Statistical analyses were performed using GraphPad Prism (v10.4.1, GraphPad) software. All data were subjected to tests for normality using the Shapiro-Wilk test or equal variance using the F-test. Comparisons for two groups that passed normality test and equal variance test were analyzed by the Student's two-tailed, unpaired *t* test. For those that passed normality but not the equal variance tests, Welsh's two-tailed, unpaired *t* test was used. Those that failed normality tests were analyzed by the nonparametric Mann-Whitney Rank Sum test. For comparisons for three or more groups, the statistical significance of parametric data was performed using a one-way ANOVA and Tukey's multiple comparison post hoc test. Comparisons for multiple groups with two independent variables were assessed using two-way ANOVA with Tukey's or Šídák's post hoc multiple comparisons test as indicated. Data are presented as mean ± SEM unless otherwise indicated. Results were considered statistically significant when *P <* 0.05.

## Results

### ApoA1 deficiency in HFD-fed *Ldlr*^KO/KO^ mice results in increased cellular apoptosis within atherosclerotic plaques

**Fig. 1:**
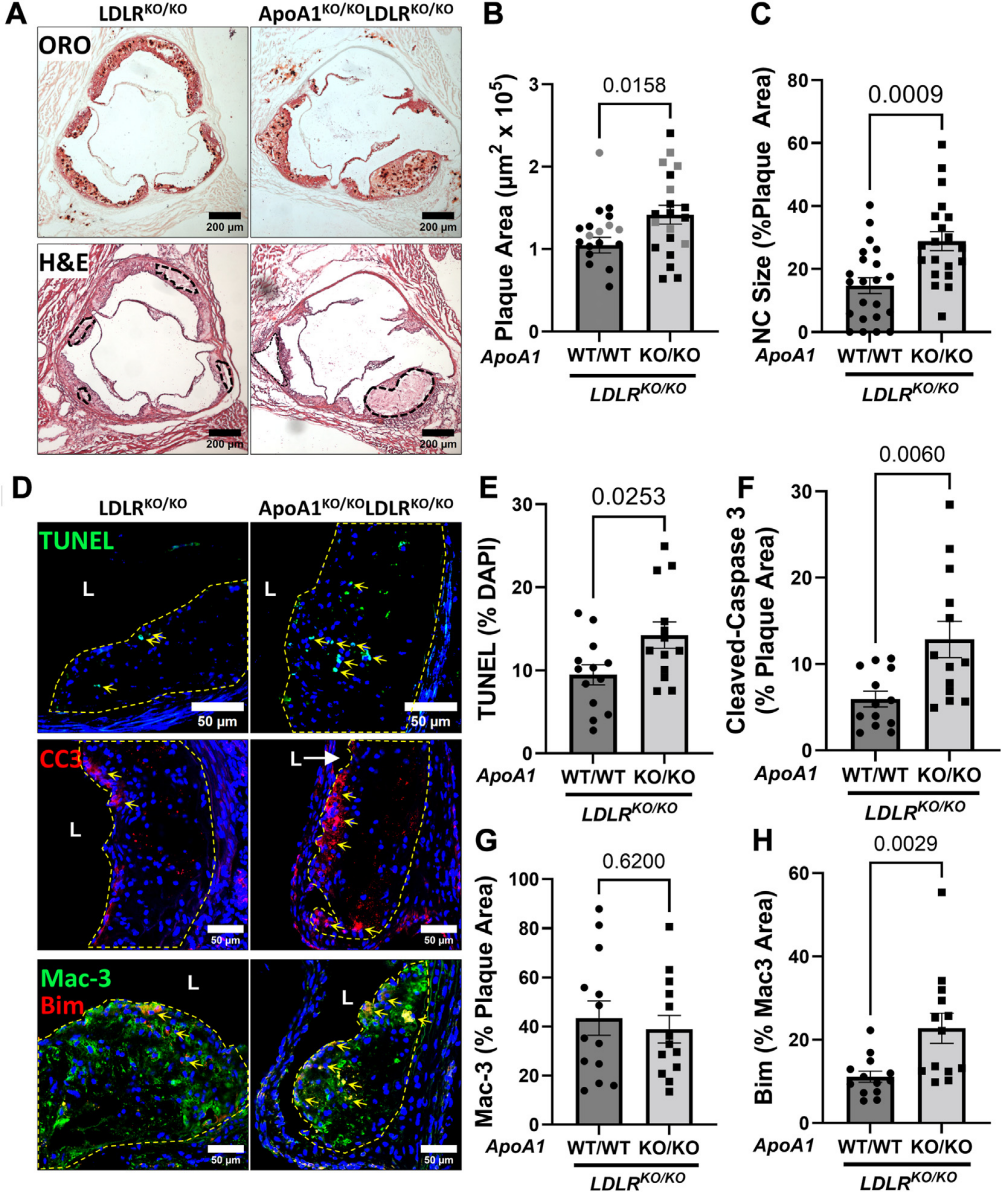
Effect of genetic deletion of ApoA1 (apolipoprotein A1) in low density lipoprotein receptor (*Ldlr*) KO mice on atherosclerotic plaque and necrotic core sizes and levels of plaque apoptosis. Male 10-week-old *ApoA1*^WT/WT^*Ldlr*^KO/KO^ (*Ldlr*^KO/KO^) and *ApoA1*^KO/KO^*Ldlr*^KO/KO^ mice (n = 22, 20) were fed a high-fat diet for 10 weeks. A: Representative images of oil red O (ORO) stained (top row), and hematoxylin and eosin (H&E) stained (bottom row) atherosclerotic plaques from aortic sinus cross sections of *Ldlr*^KO/KO^ and *ApoA1*^KO/KO^*Ldlr*^KO/KO^ mice. Dotted black line represents a necrotic core region. B: Quantification of peak plaque area taken at the apex of aortic sinus plaque profiles. Black data points represent the subset of samples used for subsequent immunofluorescent analysis. C: Quantification of necrotic core area relative to peak plaque area. D: Representative images of terminal deoxynucleotidyl transferase dUTP nick end labeling (TUNEL) labeled (top row), cleaved-caspase-3 (CC3) stained (middle row), and Mac-3 and Bim coimmunostained atherosclerotic plaques of *Ldlr*^KO/KO^ and *ApoA1*^KO/KO^*Ldlr*^KO/KO^ mice. Yellow dashed line outlines the plaque area. “L” represents the lumen of the aortic valve leaflet. E: Quantification of TUNEL-positive nuclei within the atherosclerotic plaque (n = 13, 13). F: Quantification of CC3-positive area relative to the lesional area (n = 13, 13). G: Quantification of Mac-3-positive area relative to the lesional area (n = 13, 13). H: Quantification of Bim-positive area relative to Mac-3 lesional area (n = 13, 13). For B–G: statistical analysis was conducted using an unpaired *t* test. For H: statistical analysis was conducted using Mann-Whitney test. Statistical significance is considered when *P* < 0.05. Data represent mean ± SEM. Bim, B-cell lymphoma 2 [Bcl-2] interacting mediator of cell death; CC3, cleaved caspase 3; LDLR, low-density lipoprotein receptor.

To investigate the impact of ApoA1 on necrotic core development and apoptotic cell death in atherosclerotic plaques, we placed *ApoA1*^WT/WT^*Ldlr*^KO/KO^ mice (hereafter referred to as *Ldlr*^KO/KO^ mice) and *ApoA1*^KO/KO^*Ldlr*^KO/KO^ mice on a HFD for 10 weeks. Consistent with previous studies (13), we found no significant differences in body weights between the groups after HFD feeding (supplemental Fig. S4A). As expected (13, 61), *ApoA1*^KO/KO^*Ldlr*^KO/KO^ mice had significantly lower levels of plasma total cholesterol, including free, esterified, HDL, and non-HDL cholesterol, compared to *Ldlr*^KO/KO^ mice (supplemental Fig. S4B–F). However, plasma triglyceride levels were unchanged in both groups (supplemental Fig. S4G). Histological analysis of aortic sinus cross-sections showed that *ApoA1*^KO/KO^*Ldlr*^KO/KO^ mice had larger plaque areas and necrotic cores compared to *Ldlr*^KO/KO^ mice with intact ApoA1 expression (Fig. 1A–C).

To evaluate levels of cellular apoptosis within atherosclerotic plaques, we used TUNEL to quantify DNA fragmentation, a marker of late-stage cell death (Fig. 1D, top row), and antibodies binding CC3, a key protein in the late-stage apoptosis process (Fig. 1D, middle row). We found that atherosclerotic plaques from *ApoA1*^KO/KO^*Ldlr*^KO/KO^ mice had significantly increased TUNEL-positive nuclei and CC3-positive area compared to *Ldlr*^KO/KO^ plaques (Fig. 1E, F, respectively). In contrast, overexpression of human *APOA1* in *Ldlr* KO mice resulted in dramatic reductions in atherosclerotic plaque sizes, necrotic core sizes, and reduced cellular apoptosis within atherosclerotic plaques after 10 weeks of HFD feeding (supplemental Fig. S5). Coimmunostaining from a subset of samples from *ApoA1*^WT/WT^*Ldlr*^KO/KO^ and *ApoA1*^KO/KO^*Ldlr*^KO/KO^ mice showed that CC3 positive area colocalized with macrophage staining within plaques (supplemental Fig. S6). Immunofluorescence staining for macrophages using an antibody against Mac-3 (Fig. 1D, bottom row) revealed there were no statistically significant changes in the abundance of macrophages within plaques between the *ApoA1*^WT/WT^*Ldlr*^KO/KO^ and *ApoA1*^KO/KO^*Ldlr*^KO/KO^ mice (Fig. 1G). However, immunostaining revealed higher levels of the proapoptotic protein, Bim, in macrophages within plaques of *ApoA1*^KO/KO^*Ldlr*^KO/KO^ mice (Fig. 1H).

Macrophage apoptosis in atherosclerosis can be driven by ER stress, which upregulates C/EBP homologous protein (CHOP) (62, 63). Consistent with previous reports (34), induction of ER stress in WT mouse peritoneal macrophages (MPMs) treated with increasing concentrations of TN for 8-h led to an upregulation of Bim expression (supplemental Fig. S7A, B). However, we observed no differences in the levels of ER stress markers GRP78 or CHOP in cultured peritoneal macrophages challenged with ER stress-inducing agents 7-ketocholesterol (7KC) or TN in the absence or presence of HDL (supplemental Fig. S7C–E) or in vivo in atherosclerotic plaques of HFD-fed *ApoA1*^KO/KO^*Ldlr*^KO/KO^ versus *ApoA1*^WT/WT^*Ldlr*^KO/KO^ mice (supplemental Fig. S7F–H). Therefore, the increased level of Bim protein in atherosclerotic plaque macrophages in *ApoA1*^KO/KO^*Ldlr*^KO/KO^ mice is not the consequence of elevated ER stress in those cells compared to those of *ApoA1*^WT/WT^*Ldlr*^KO/KO^ mice.

### Bim-deficient macrophages are protected against TN-induced macrophage apoptosis

**Fig. 2:**
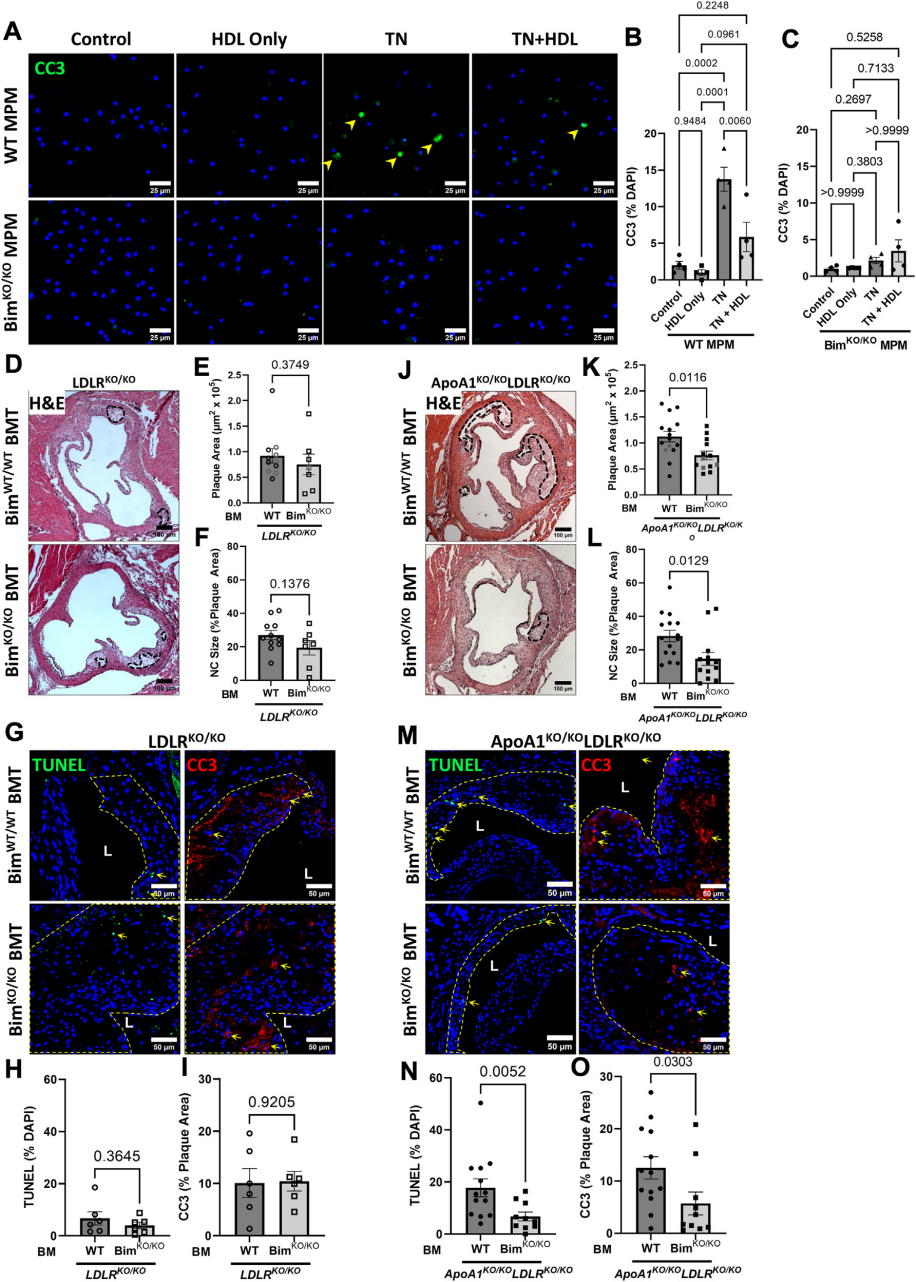
Bim deletion in bone marrow of atherosclerotic *ApoA1*^KO/KO^*Ldlr*^KO/KO^ mice reduces atherosclerotic plaque, necrotic core sizes, and plaque apoptosis. Thioglycolate-elicited mouse peritoneal macrophages (MPM) from wild-type (WT) C57/BL6 and *Bim*^KO/KO^ mice were harvested and cultured in media containing lipoprotein-deficient serum and treated with or without 50 μg/ml human high-density lipoprotein (HDL) and with or without 10 μg/ml tunicamycin (TN). A: Representative images of CC3 stained peritoneal macrophages from WT mice (top row) and *Bim*^KO/KO^ mice (bottom row). B, C: Quantification of CC3 stained MPMs from WT mice and *Bim*^KO/KO^ mice, respectively (n = 4, 4). Isolated bone marrow from WT mice and *Bim*^KO/KO^ mice were transplanted into lethally irradiated male 10-week-old *Ldlr*^KO/KO^. Following a 4-week recovery period post transplantation, transplanted mice were fed a high-fat diet (HFD) for 10 weeks. D: Representative images of hematoxylin and eosin (H&E) stained atherosclerotic plaques from transplanted *Ldlr*^KO/KO^ mice. Dotted black line represents the necrotic core region. Analysis of E: peak plaque area, and F: relative necrotic core from transplanted *Ldlr*^KO/KO^ mice (n = 11, 7). Black data points represent the subset of samples used for subsequent immunofluorescent analysis. G: Representative images of TUNEL (left column) and CC3 stained (right column) atherosclerotic plaques of transplanted *Ldlr*^KO/KO^ mice. Yellow dashed line outlines the plaque area. “L” represents the lumen of the aortic valve leaflet. H: Quantification of TUNEL-positive nuclei within the atherosclerotic plaque (n = 6, 6). I: Quantification of CC3-positive area relative to the lesional area (n = 6, 6). Male 10-week-old *ApoA1*^KO/KO^*Ldlr*^KO/KO^ mice were transplanted with BM from WT or *Bim*^KO/KO^ donors and fed a HFD for 10 weeks in a similar fashion as above *Ldlr*^KO/KO^ mice. J: Representative images of H&E-stained atherosclerotic plaques from transplanted *ApoA1*^KO/KO^*Ldlr*^KO/KO^ mice. Analysis of K: peak plaque area, and L: relative necrotic core from transplanted *ApoA1*^KO/KO^*Ldlr*^KO/KO^ mice (n = 15, 13). M: Representative images of TUNEL labeled (left) and CC3 stained (right) atherosclerotic plaques of transplanted *ApoA1*^KO/KO^*Ldlr*^KO/KO^ mice. N: Quantification of TUNEL-positive nuclei within the atherosclerotic plaque (n = 13, 10). O: Quantification of CC3-positive area relative to the lesional area (n = 13, 10). For B, C: statistical analysis was conducted using one-way ANOVA with Tukey's post hoc multiple comparisons test. For E, L, O: statistical analysis was conducted using Mann Whitney test. For F–K and N: statistical analysis was conducted using an unpaired *t* test. Statistical significance is considered when *P* < 0.05. Data represent mean ± SEM. ApoA1, apolipoprotein A1; Bim, B-cell lymphoma 2 [Bcl-2] interacting mediator of cell death; CC3, cleaved caspase 3; LDLR, low-density lipoprotein receptor; MPM, mouse peritoneal macrophage.

We have previously reported that HDL treatment of cultured MPMs protects them against apoptosis induced by a variety of stressors, including those inducing ER stress (45). HDL treatment protected MPMs from WT mice against apoptosis induced by TN treatment, as measured by immunofluorescent staining for CC3 (Fig. 2A, B and supplemental Fig. S8A). On the other hand, treatment with the same protein concentration of lipid-free ApoA1 did not protect MPMs from WT mice against apoptosis induced by TN (supplemental Fig. S8C, D). MPMs from *Bim*^KO/KO^ mice were protected to a similar degree against TN-induced macrophage apoptosis (Fig. 2A, C and supplemental Fig. S8B), and HDL treatment provided no further protection (Fig. 2A, C and supplemental Fig. S8B). HDL has been reported to protect macrophages against apoptosis by mediating sterol efflux (64, 65); however, *Bim* KO did not impact the rate of either HDL- or ApoA1-mediated sterol efflux from cultured macrophages (supplemental Fig. S9). These data lead us to hypothesize that deletion of Bim may compensate for the effect of reduced HDL due to deletion of ApoA1 on apoptosis in atherosclerotic plaques in *Ldlr*^KO/KO^ mice.

### Bone marrow selective deficiency of Bim reduces plaque size and plaque apoptosis in *ApoA1*^KO/KO^*Ldlr*^KO/KO^ mice

To examine the impact of Bim on atherosclerosis, *Ldlr*^KO/KO^ mice were transplanted with BM from WT mice (WT BMT) or *Bim*^KO/KO^ mice (*Bim*^KO/KO^ BMT) and fed a HFD for 10 weeks. Atherosclerotic plaques were analyzed in stained aortic sinus cross sections (Fig. 2D). In BM-transplanted *Ldlr*^KO/KO^ mice, there were no significant differences in plaque sizes (Fig. 2E and supplemental Fig. S10A, B) or the necrotic core sizes within atherosclerotic plaques between mice that received WT BM and *Bim*^KO/KO^ BM (Fig. 2F). Similarly, there were no differences in the extent of apoptosis within atherosclerotic plaques as revealed by TUNEL and CC3 staining (Fig. 2G–I). In a separate experiment, male *ApoA1*^KO/KO^*Ldlr*^KO/KO^ mice were transplanted with BM from either WT or *Bim*^KO/KO^ donors and fed HFD for 10 weeks. In these mice, a significant reduction in plaque sizes were observed with mice transplanted with *Bim*^KO/KO^ BM compared with WT BM (Fig. 2J, K and supplemental Fig. S10C, D). Furthermore, there were significant decreases in the necrotic core area relative to the plaque area in *ApoA1*^KO/KO^*Ldlr*^KO/KO^ mice transplanted with *Bim*^KO/KO^ BM (Fig. 2L) as well as in levels of apoptosis measured by TUNEL and CC3 staining (Fig. 2M–O). We have recently reported that ApoA1 deficiency increased necroptosis in macrophages in atherosclerotic plaques in vivo and HDL treatment of macrophages in vitro protected them from necroptosis (13). However, we saw no impact of inactivation of Bim in macrophages on their susceptibility to induction of necroptosis in culture (supplemental Fig. S11A, B), or of inactivation of Bim in BM derived cells on the levels of phosphorylated mixed lineage kinase domain-like protein (pMLKL), a marker of necroptosis induction, in atherosclerotic plaques in BM transplanted ApoA1^KO/KO^LDLR^KO/KO^ mice (supplemental Fig. S11C, D), indicating that, as expected, inactivation of Bim in BM-derived cells specifically impacts the apoptotic death pathway in atherosclerotic plaques of *ApoA1*^KO/KO^*Ldlr*^KO/KO^ mice.

### *Bim*^KO/KO^ BM transplanted mice develop splenomegaly with increased circulating leukocytes and have diminished levels of plasma lipids

**Fig. 3:**
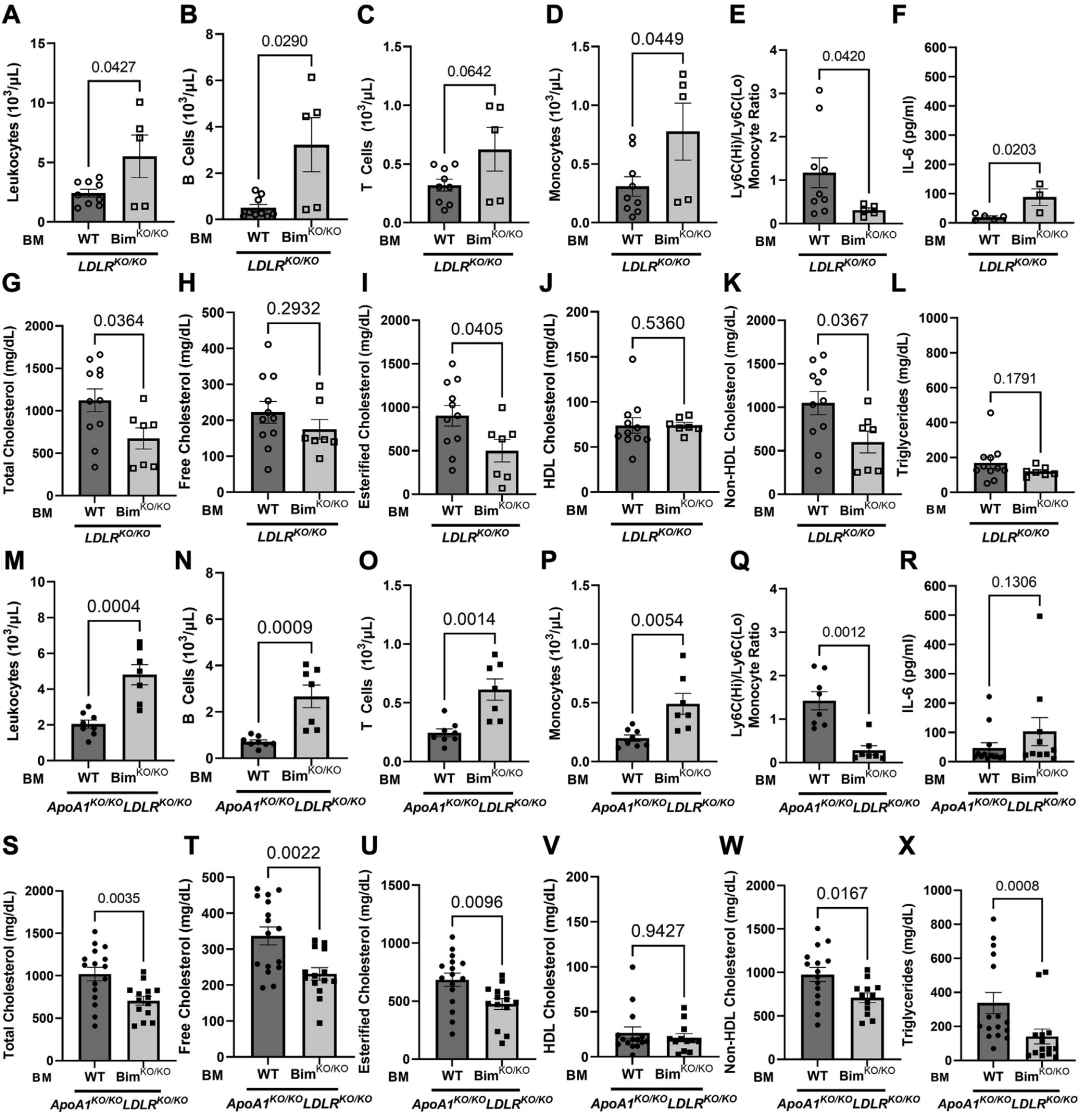
Bim deletion in bone marrow-derived cells of atherosclerotic mice reduces plasma cholesterol and increases circulating leukocytes. Peripheral blood was collected by cardiac puncture from 10-week HFD-fed WT and *Bim*^KO/KO^ BMT mice fasted overnight. Absolute concentrations of (A) leukocytes; (B) B cells; (C) T cells; and (D) monocytes in peripheral blood samples of LDLR^KO/KO^ BMT mice (n = 9, 5) measured by flow cytometry. Absolute concentrations of (M) leukocytes; (N) B cells; (O) T cells; and (P) monocytes in peripheral blood of *ApoA1*^KO/KO^*Ldlr*^KO/KO^ BMT mice (n = 8, 7). E and Q, Ratio of Ly6C High (Hi) monocytes to Ly6C Low (Lo) monocytes in *Ldlr*^KO/KO^ BMT and *ApoA1*^KO/KO^*Ldlr*^KO/KO^ BMT mice, respectively. F and R, Plasma IL-6 concentration in *Ldlr*^KO/KO^ BMT (n = 5, 3) and *ApoA1*^KO/KO^*Ldlr*^KO/KO^ BMT mice (n = 13, 10), respectively. Lipids were analyzed by performing colorimetric enzymatic assays on plasma samples isolated from collected blood. Quantification of G, total; H, free; I, esterified; J: HDL, K: non-HDL cholesterol; and L, triglycerides from plasma of *Ldlr*^KO/KO^ BMT mice (n = 11, 7). Quantification of S, total; T, free; U, esterified; V, HDL, W, non-HDL cholesterol; and X, triglycerides from plasma of *ApoA1*^KO/KO^*Ldlr*^KO/KO^ BMT mice (n = 16, 14). For A, C, D, F–I, K, M–P, S–U, and W, statistical analysis was conducted using unpaired *t* test. For B, E, J, L, Q, R, V, and X: statistical analysis was conducted using Mann Whitney test. Statistical significance is considered when *P* < 0.05. Data represent mean ± SEM. Bim, B-cell lymphoma 2 [Bcl-2] interacting mediator of cell death; BMT, bone marrow transplantation; HFD, high-fat diet; IL-6, interleukin-6; LDLR, low-density lipoprotein receptor.

Previous reports have shown that KO of Bim, either globally or specifically in BM-derived cells in mice, leads to splenomegaly and expansion of blood lymphoid and myeloid cells as a result of impaired leukocyte apoptosis (44, 66). Consistent with those reports, both *Ldlr*^KO/KO^ and *ApoA1*^KO/KO^*Ldlr*^KO/KO^ mice transplanted with BM from *Bim*^KO/KO^ donors exhibited splenomegaly (but no other differences in body or organ weights measured; supplemental Fig. S12) and increased blood leukocytes (Fig. 3A, M) when compared with mice transplanted with BM from WT donors.

Similarly, we observed increases or trends toward increases in B-cells, T-cells, and monocytes and reductions in the ratios of Ly6C-High (Hi) to Ly6C-Low (Lo) monocytes (driven by increases in Ly6C-Lo monocytes) in both *Ldlr*^KO/KO^ and *ApoA1*^KO/KO^ *Ldlr*^KO/KO^ mice transplanted with BM from *Bim*^KO/KO^ compared to WT donors (Fig. 3B–E, N–Q and supplemental Fig. S13). In *Ldlr*^KO/KO^ recipients, transplantation of *Bim*^KO/KO^ BM increased plasma IL-6 levels (Fig. 3F). A similar trend in the average plasma IL-6 levels was observed in *ApoA1*^KO/KO^*Ldlr*^KO/KO^ recipients, although the data did not reach statistical significance (Fig. 3R). Despite this, there were no differences in the extent of IL-6 staining in atherosclerotic plaques (supplemental Fig. S14A–D).

Analysis of plasma lipids revealed that transplantation with BM from *Bim*^KO/KO^ donors was accompanied by reduced plasma total and esterified cholesterol (Fig. 3G, I, S, U) in both *Ldlr*^KO/KO^ and *ApoA1* ^KO/KO^*Ldlr*^KO/KO^ mice compared to those transplanted with BM from WT donors. Transplanting *Bim*^KO/KO^ BM did not reduce free cholesterol (Fig. 3H) or triglyceride concentrations (Fig. 3J) in *Ldlr*^KO/KO^ mice but did in *ApoA1*^KO/KO^*Ldlr*^KO/KO^ mice (Fig. 3T, X). Recipients transplanted with BM from *Bim*^KO/KO^ donors did not exhibit altered HDL cholesterol but did exhibit reduced non-HDL cholesterol concentrations compared to WT BM donors (Fig. 3J, K, V, W). Transplanting *Bim*^KO/KO^ BM did not impair triglyceride secretion in fasted *Ldlr*^KO/KO^ mice treated with the lipoprotein lipase inhibitor tyloxapol, a standard measure of hepatic VLDL secretion (58, 59) (supplemental Fig. S15).

### Inactivation of Bim in BM-derived myeloid cells reduces plaque apoptosis, necrotic core, and atherosclerotic plaque sizes in ApoA1^KO/KO^ LDLR^KO/KO^ mice

We observed that apoptosis staining in atherosclerotic plaques was primarily associated with macrophages (supplemental Fig. S6); therefore, we tested the impact of inactivating Bim in BM-derived myeloid cells. To do this, we crossed mice engineered so that the gene encoding Bim contained loxP recombination sites (*Bim*^fl/fl^ mice) (55) and mice in which the bacterial Cre-recombinase was knocked into the lysozyme M (LyzM) gene (*LyzM*^cre/cre^) such that Cre is expressed in myeloid-derived cells including macrophages (67). Immunoblotting and flow cytometric analysis of peritoneal macrophages isolated from *LyzM*^cre/cre^*Bim*^fl/fl^ mice (*Bim*^MKO^) and control *LyzM*^cre/cre^*Bim*^WT/WT^ mice (*Bim*^MWT^) confirmed the absence of Bim protein in macrophages from *Bim*^MKO^ mice (supplemental Fig. S16).

**Fig. 4:**
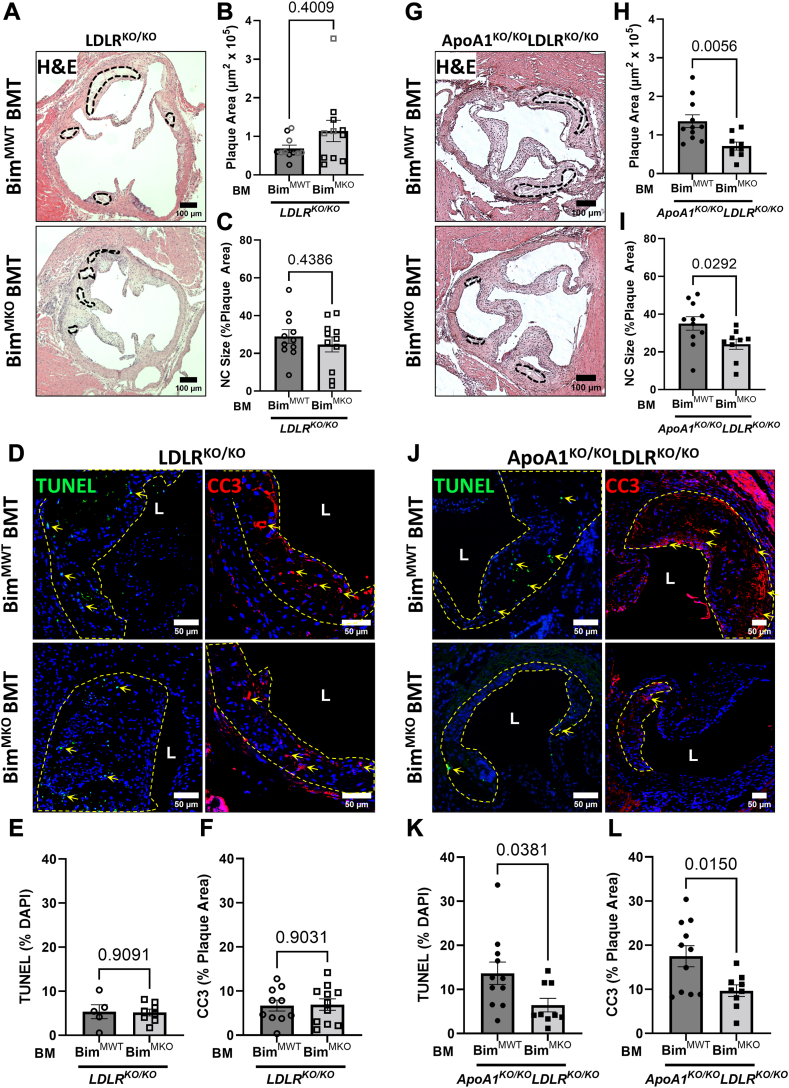
Deletion of myeloid Bim reduces atherosclerotic plaque, necrotic core area, and plaque apoptosis in atherosclerotic plaques of *ApoA1*^KO/KO^*Ldlr*^KO/KO^ mice. Male 10-week-old *Ldlr*^KO/KO^ were transplanted with bone marrow (BM) from *LyzM*^cre/cre^ (*Bim*^MWT^) or *LyzM*^cre/cre^*Bim*^fl/fl^ (*Bim*^MKO^) donors and fed a high-fat diet (HFD) for 10 weeks. A: Representative images of hematoxylin and eosin (H&E) stained atherosclerotic plaques from transplanted *Ldlr*^KO/KO^ mice. Dotted black line represents the necrotic core region. Analysis of B: peak plaque area and C: relative necrotic core from transplanted *Ldlr*^KO/KO^ mice (n = 11, 11). Black data points represent the subset of samples used for subsequent immunofluorescent analysis. D: Representative images of TUNEL labeled (left) and CC3 stained (right) atherosclerotic plaques of transplanted *Ldlr*^KO/KO^ mice. Yellow dashed line outlines the plaque area. “L” represents the lumen of the aortic valve leaflet. E: Quantification of TUNEL-positive nuclei within the atherosclerotic plaque (n = 5, 8). F: Quantification of CC3-positive area relative to the lesional area (n = 10, 11). Male 10-week-old *ApoA1*^KO/KO^*Ldlr*^KO/KO^ mice were transplanted with BM from *Bim*^MWT^ or *Bim*^MKO^ donors and fed a HFD for 10 weeks. G: Representative images of H&E-stained atherosclerotic plaques from transplanted *ApoA1*^KO/KO^*Ldlr*^KO/KO^ mice. Analysis of H: peak plaque area and I, relative necrotic core from transplanted *ApoA1*^KO/KO^*Ldlr*^KO/KO^ mice (n = 11, 9). J: Representative images of TUNEL labeled (left) and CC3 stained (right) atherosclerotic plaques of transplanted *ApoA1*^KO/KO^*Ldlr*^KO/KO^ mice. K: Quantification of TUNEL-positive nuclei within the atherosclerotic plaque (n = 11, 9). L: Quantification of CC3-positive area relative to the lesional area (n = 11, 9). For C–I, and L: statistical analysis was conducted using unpaired *t* test. For B, K: statistical analysis was conducted using Mann-Whitney test. Statistical significance is considered when *P* < 0.05. Data represents mean ± SEM. Bim, B-cell lymphoma 2 [Bcl-2] interacting mediator of cell death; CC3, cleaved caspase 3; LDLR, low-density lipoprotein receptor.

In separate experiments, *Ldlr*^KO/KO^ or *ApoA1*^KO/KO^*Ldlr*^KO/KO^ mice were transplanted with BM from either *Bim*^MKO^ or control *Bim*^MWT^ donors and fed the HFD for 10 weeks after which atherosclerosis was analyzed. Transplantation of *Ldlr*^KO/KO^ mice with BM from *Bim*^MKO^ donors did not significantly affect atherosclerotic plaque sizes, necrotic core sizes (Fig. 4A–C and supplemental Fig. S17A, B), or levels of apoptosis within atherosclerotic plaques (Fig. 4D–F). Female *Ldlr*^KO/KO^ mice transplanted with either *Bim*^MWT^ or *Bim*^MKO^ BM also exhibited no differences in atherosclerotic plaque sizes or necrotic core sizes when they were fed the HFD for 10 weeks (supplemental Fig. S18). When the feeding time was extended to 20 weeks, necrotic core sizes were enlarged in the BM-transplanted female *Ldlr*^KO/KO^ mice; however, again transplantation with *Bim*^MKO^ BM did not impact either plaque or necrotic core sizes (supplemental Fig. S18). In contrast, when *ApoA1*^KO/KO^*Ldlr*^KO/KO^ mice were transplanted with BM from *Bim*^MKO^ versus *Bim*^MWT^ donors and fed the HFD for 10 weeks, *Bim* KO in BM-derived myeloid cells resulted in significantly smaller atherosclerotic plaques, necrotic core sizes (Fig. 4G–I and supplemental Fig. S17C, D), and levels of apoptosis within atherosclerotic plaques (Fig. 4J–L).

### Bim^MKO^ BM transplanted mice do not exhibit elevated blood leukocyte counts or plasma cholesterol levels

**Fig. 5:**
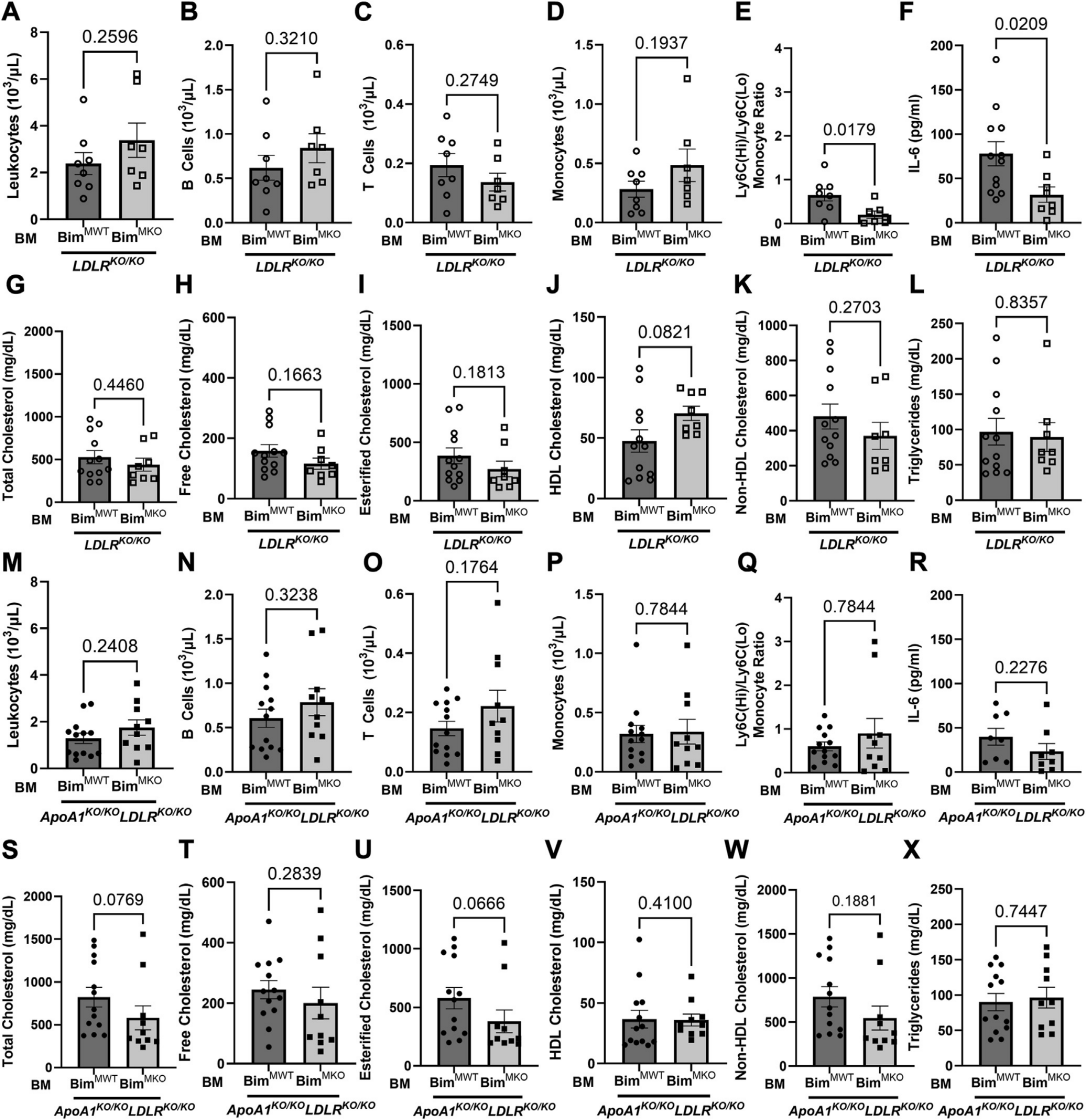
Myeloid-specific Bim deletion in bone marrow of atherosclerotic mice does not affect plasma cholesterol levels or circulating leukocyte concentrations. Peripheral blood was collected by cardiac puncture from 10-week HFD-fed *Bim*^MWT^ and *Bim*^MKO^ BMT mice fasted overnight. Absolute concentrations of A: leukocytes; (B) B cells; (C) T cells; and (D) monocytes in peripheral blood samples of *Ldlr*^KO/KO^ BMT mice (n = 8, 7) measured by flow cytometry. Absolute concentrations of (M) leukocytes; (N) B cells; (O) T cells; and (P) monocytes in peripheral blood samples of *ApoA1*^KO/KO^*Ldlr*^KO/KO^ BMT mice (n = 13, 10). E, Q: Ratio of Ly6C High (Hi) monocytes to Ly6C Low (Lo) monocytes in *Ldlr*^KO/KO^ BMT and *ApoA1*^KO/KO^*Ldlr*^KO/KO^ BMT mice, respectively. F and R: Plasma IL-6 concentration in *Ldlr*^KO/KO^ BMT (n = 12, 8) and *ApoA1*^KO/KO^*Ldlr*^KO/KO^ BMT mice (n = 8, 8), respectively. Lipids were analyzed by performing colorimetric enzymatic assays on plasma samples isolated from collected blood. Quantification of (G) total; (H) free; (I) esterified; (J) HDL, (K) non-HDL cholesterol; and (L) triglycerides from plasma of *Ldlr*^KO/KO^ BMT mice (n = 12, 8). Quantification of (S) total; (T) free; (U) esterified; (V) HDL, (W) non-HDL cholesterol; and (X) triglycerides from plasma of *ApoA1*^KO/KO^*Ldlr*^KO/KO^ BMT mice (n = 13, 10). For A–H, J, M–O, R, and X: statistical analysis was conducted using unpaired *t* test. For I, K, L, P, Q, and S–W: statistical analysis was conducted using Mann-Whitney test. Statistical significance is considered when *P* < 0.05. Data represent mean ± SEM. Bim, B-cell lymphoma 2 [Bcl-2] interacting mediator of cell death; BMT, bone marrow transplantation; IL-6, interleukin-6; HFD, high-fat diet; LDLR, low-density lipoprotein receptor.

No differences were observed in body weights or liver to body weight ratios in all groups (supplemental Fig. S19). However, heart/body weight ratios were slightly reduced in *Ldlr*^KO/KO^ but not *ApoA1*^KO/KO^*Ldlr*^KO/KO^ mice transplanted with *Bim*^MKO^ compared to *Bim*^MWT^ BM (supplemental Fig. S19B, F) and spleen/body weight ratios were increased in *ApoA1*^KO/KO^*Ldlr*^KO/KO^ but not in *Ldlr*^KO/KO^ mice transplanted with *Bim*^MKO^ compared to *Bim*^MWT^ BM (supplemental Fig. S19D, H). There were no significant differences in concentrations of circulating leukocytes, including T-cells, B-cells, or monocytes when either *Ldlr*^KO/KO^ or *ApoA1*^KO/KO^*Ldlr*^KO/KO^ mice were transplanted with BM from either *Bim*^MKO^ or control *Bim*^MWT^ donors (Fig. 5A–E, M–Q and supplemental Fig. S20). Plasma IL-6 levels were significantly lower in LDLR^KO/KO^ mice transplanted with Bim^MKO^ BM (Fig. 5F) and exhibited a trend (not statistically significant) to be lower in *ApoA1*^KO/KO^*Ldlr*^KO/KO^ mice transplanted with Bim^MKO^ BM (Fig. 5P). Despite this, there were no statistically significant differences in levels of IL-6 detected in atherosclerotic plaques (supplemental Fig. S14E–H). Furthermore, no differences were detected in abundance of leukocytes or macrophages in atherosclerotic plaques from either *Ldlr*^KO/KO^ or from *ApoA1*^KO/KO^*Ldlr*^KO/KO^ mice that were transplanted with BM from either WT or *Bim*^KO/KO^ mice or from *Bim*^MWT^ or *Bim*^MKO^ mice after 10 weeks of HFD feeding supplemental Fig. S21).

There were no statistically significant effects on plasma total cholesterol, free cholesterol, esterified cholesterol, HDL cholesterol, non-HDL cholesterol, or triglycerides when either *Ldlr*^KO/KO^ (Fig. 5G–L) or *ApoA1*^KO/KO^*Ldlr*^KO/KO^ mice (Fig. 5S–X) were transplanted with BM from either *Bim*^MKO^ compared to control *Bim*^MWT^ donors. Therefore, Bim expressed in myeloid cells does not appear to impact plasma cholesterol levels, suggesting that the reduced plasma cholesterol levels observed when Bim was inactivated in all BM-derived cells likely reflected the effects of inactivation of Bim in one or more nonmyeloid cell types derived from BM. These data demonstrate that the selective inactivation of Bim in myeloid cells is sufficient to counteract the increased apoptosis, necrotic core, and atherosclerotic plaque size resulting from ApoA1 deficiency in *ApoA1*^KO/KO^*Ldlr*^KO/KO^ mice, even though it does not impact the level of plaque apoptosis, necrotic core development, or plaque size in mice expressing normal levels of ApoA1.

## Discussion

Cellular apoptosis in atherosclerotic plaques plays a dual role in plaque development, limiting early stages of plaque growth, when pathways for efferocytosis are intact and efficiently clear apoptotic corpses, but contributing to more advanced stages of plaque growth and necrotic core formation when pathways for efferocytosis are impaired (68, 69, 70, 71, 72). In this study, we demonstrate that deficiency of ApoA1 increases the extent of apoptosis of macrophages within atherosclerotic plaques of experimental mice. We have previously reported that deletion of *ApoA1* gene expression similarly increases the extent of programmed necrosis in macrophages within atherosclerotic plaques (13). Our current and previous findings suggest that ApoA1 attenuates both apoptosis and programmed necrosis of macrophages and thereby attenuates necrotic core and atherosclerotic plaque growth (13, 45, 46). Analysis of macrophages in culture suggests that this effect may be due to HDL, which is reduced when ApoA1 expression is eliminated, since HDL mediates protection of cultured macrophages against the induction of apoptotic and necroptotic cell death pathways (13, 45, 46). While we cannot rule out the possibility that reduced macrophage apoptosis within atherosclerotic plaques may be the consequence of reductions in non-HDL cholesterol that are also seen in the *ApoA1*^KO/KO^*Ldlr*^KO/KO^ mice, we think this unlikely because we have previously reported that HDL, but not LDL, protects macrophages from apoptosis induced by a variety of stressors (45, 46). We demonstrate that eliminating ApoA1 increases the abundance of Bim protein in atherosclerotic plaques of *Ldlr* KO mice and show that *Bim* KO in BM-derived myeloid cells reverses the increased apoptosis, necrotic core development, and atherosclerotic plaque size resulting from ApoA1 deficiency in *ApoA1*^KO/KO^*Ldlr*^KO/KO^ mice.

In contrast, inactivation of Bim in BM-derived myeloid cells did not impact apoptosis or necrotic core sizes in atherosclerotic plaques of mice with intact ApoA1 expression (*Ldlr*^KO/KO^ mice). One potential explanation may be that the plaques that developed over the 10-week HFD feeding period in the *Ldlr*^KO/KO^ mice were smaller, with smaller necrotic cores, representing less advanced stages than those in the *ApoA1*^KO/KO^*Ldlr*^KO/KO^ mice. However, when we extended the feeding time for *Ldlr*^KO/KO^ mice to 20 weeks to induce plaques with larger necrotic cores, we, again saw no effect of *Bim* KO (supplemental Fig. S18). These observations suggest that the ability of *Bim* KO in myeloid cells to reduce plaque apoptosis and necrotic core sizes reflects the absence of ApoA1 rather than the stage of atherosclerotic plaque development. Therefore, our findings that (1) the absence of ApoA1 results in increased levels of Bim protein, macrophage apoptosis, and necrotic core development and the sizes of atherosclerotic plaques and (2) that Bim inactivation in BM-derived myeloid cells reduces macrophage apoptosis, necrotic core development and the sizes of atherosclerotic plaques of *ApoA1*^KO/KO^*Ldlr*^KO/KO^ mice, suggest that ApoA1 may counteract necrotic core development in atherosclerotic plaques at least in part by suppressing macrophage apoptosis through suppression of Bim protein levels.

HDL has been reported to protect macrophages against apoptosis by mediating ABCG1 dependent efflux of oxysterols (64, 65) and inactivation of ABCG1 in BM-derived cells was associated with increased apoptosis but reduced atherosclerotic plaque sizes with longer high fat diet feeding times (64, 73). These findings appear to contradict our observation of increased plaque and necrotic core sizes with ApoA1 inactivation in *Ldlr*^KO/KO^ mice. However, ABCG1 deficiency in myeloid cells has been associated with increased levels of efferocytosis activity (74). On the other hand, we previously reported that the presence or absence of HDL did not affect efferocytosis of apoptotic corpses by mouse macrophages, at least in culture (45). This may explain the different effects of global ApoA1 KO versus myeloid ABCG1 KO on atherosclerotic plaque and necrotic core sizes even though both are expected to similarly impair HDL-mediated cholesterol efflux and induce apoptosis. We previously reported that HDL protects against apoptosis and necroptosis by activating the PI3K-Akt1 survival signaling pathway in macrophages and other cell types (13, 45, 75). Whether this is secondary to or independent of sterol efflux is not yet clear. Regardless of the mechanism, however, we demonstrate that Bim KO can circumvent the increased apoptosis resulting from absence of HDL in vitro or KO of ApoA1 in vivo even though Bim deficiency in myeloid cells does not impact either HDL or ApoA1 stimulated efflux. Rather, this is likely the result of Bim's central role in promoting the intrinsic apoptosis pathway (34).

Targeting Bim in myeloid cells appears to compensate for the increased apoptosis and necrotic core development resulting from ApoA1 inactivation and resulting HDL deficiency. HDL deficiency in humans, though rare, can result from homozygous or compound heterozygous loss-of-function mutations affecting *APOA1*, *ABCA1*, or lecithin:cholesterol acyltransferase and are often accompanied by premature atherosclerotic disease (76, 77). HFD fed *ApoE* KO mice also exhibit low HDL cholesterol. Whether targeting Bim can alleviate apoptosis and necrotic core development within atherosclerotic plaques in the context of other states of low HDL cholesterol, or whether this effect is specific for ApoA1 deficiency, remains to be determined.

Both the *Ldlr*^KO/KO^ and the *ApoA1*^KO/KO^*Ldlr*^KO/KO^ mice transplanted with *Bim*^KO/KO^ BM exhibited splenomegaly, increased circulating T-lymphocytes and monocytes and reduced Ly6C-Hi/Lo monocyte ratios compared to mice transplanted with WT BM. This is consistent with Bim's critical role in maintaining leukocyte hemostasis and preventing autoimmune cell maturity (44, 66, 78, 79). We also observed increased plasma IL-6 levels in *Ldlr*^KO/KO^ recipients transplanted with *Bim*^KO/KO^ BM. However, neither levels of IL-6 nor the abundance of leukocytes in atherosclerotic plaques were impacted by transplantation of BM from either *Bim*^KO/KO^ or *Bim*^MKO^ compared to control donors into either *Ldlr*^KO/KO^ or *ApoA1*^KO/KO^*Ldlr*^KO/KO^ recipients. This suggests that factors other than systemic IL-6 levels were more impactful on atherosclerotic plaque development in these mice. Consistent with prior reports, we also observed reduced plasma cholesterol levels in *Ldlr*^KO/KO^ (66), and in *ApoA1*^KO/KO^ *Ldlr*^KO/KO^ recipients transplanted with BM from *Bim*^KO/KO^ compared to WT donors. This does not appear to be due to altered hepatic production of VLDL, at least in the *Ldlr*^KO/KO^ recipients, since there were no differences in triglyceride secretion rates. The mechanisms remain unclear, but they do not appear to be responsible for the reduced atherosclerotic plaque and necrotic core sizes when Bim was inactivated in BM-derived cells in *ApoA1*^KO/KO^*Ldlr*^KO/KO^ mice. This is because inactivation of Bim in BM-derived myeloid cells in *ApoA1*^KO/KO^*Ldlr*^KO/KO^ exhibited the same reductions in atherosclerotic plaque sizes, necrotic core sizes and apoptosis levels without impacting plasma lipoprotein associated cholesterol or TG levels. Thus, the reductions in plasma cholesterol were not driven by Bim inactivation in myeloid cells. The extent to which the increased circulating B- and T-lymphocytes and monocytes resulting from global Bim KO in BM derived cells was responsible for the reduced plasma cholesterol levels remains to be determined. However, it is interesting to note that hypocholesterolemia has been reported in patients with hematological malignancies, although the mechanisms have not been established (80, 81, 82, 83).

Our findings suggest that the increased cellular apoptosis in atherosclerotic plaques observed in *ApoA1*^KO/KO^*Ldlr*^KO/KO^ mice was dependent on Bim expression in myeloid cells, and this is consistent with the increased levels of Bim in atherosclerotic plaque macrophages observed in those mice. It is also consistent with observations that HDL and Bim inactivation similarly protected cultured macrophages from apoptosis induced by TN, a potent inducer of ER stress. These results are consistent with previous studies showing that inactivation of Bim protected against macrophage apoptosis in response to oxLDL, growth factor withdrawal, and thapsigargin (34, 44, 66).

The mechanisms by which ApoA1 deficiency increases Bim protein levels in myeloid cells in atherosclerotic plaques remain unclear. The extent to which this is driven by impaired cholesterol efflux remains to be tested. Our group previously demonstrated that HDL can protect macrophages against apoptotic and necroptotic cell death through a pathway dependent on the HDL receptor, SR-BI, and PDZK1, and involving activation of PI3K/Akt signaling pathways (13, 45). In addition, others have reported that HDL can activate mitogen-activated protein kinase (MAPK), extracellular signal-regulated kinases (ERK), and Janus kinase (JAK)-signal transducer and activator of transcription (STAT) signaling pathways in different cell types (84, 85, 86, 87, 88, 89), and many of these have been reported to suppress Bim at the level of gene expression post translational regulation (90, 91). Our findings suggest that this is not through the regulation of CHOP induction. However, the mechanism by which ApoA1 attenuates Bim protein levels in macrophages within atherosclerotic plaques remains unclear, highlighting an area for potential future research into the functions of ApoA1 and HDL.

Our findings suggest Bim in myeloid cells as a potential target for inhibition to protect against atherosclerosis development in the context of ApoA1 deficiency. Whether this applies to other conditions of very low HDL levels (such as ABCA1 or lecithin:cholesterol acyltransferase deficiency) remains to be tested. Whether targeting Bim in myeloid cells may be effective against preestablished atherosclerotic plaques is also not clear, since our studies examined new atherosclerotic plaque development in the context of Bim targeting. Finally, targeting Bim may also have undesirable effects. For example, inactivation of Bim in BM is accompanied by expansion of circulating lymphoid and myeloid cell populations, and autoimmune disorders (44, 90). Bim is also found deleted in a number of malignancies, including certain lymphomas and plays an important tumor suppressive role in cancer (90, 91, 92). Whether these can be avoided by more selective targeting of Bim in atherosclerotic plaque macrophages remains to be investigated.

## Data availability

The data described in the manuscript are available in the Supplementary materials or from B.

Trigatti, McMaster University (trigatt@mcmaster.ca) upon request.

## Supplemental data

This article contains supplemental data.

## Conflict of interest

The authors declare that they have no conflicts of interest with the contents of this article.
