# Supplementary material for: Apolipoprotein A1 deficiency increases macrophage apoptosis and necrotic core development in atherosclerotic plaques in a Bim-dependent manner
Source: J Lipid Res. 2025 Mar 20;66(5):100782. doi: 10.1016/j.jlr.2025.100782 (PMC12051063; doi:10.1016/j.jlr.2025.100782)
Supplement: Supplemental Figures [file mmc2.pdf]

Apolipoprotein A1 deficiency increases Macrophage Apoptosis and Necrotic Core Development in Atherosclerotic Plaques in a Bim dependent manner.

Alexander S. Qian, George E G Kluck, Pei Yu, Leticia Gonzalez, Elizabeth Balint, and Bernardo L Trigatti

Supplemental Figures

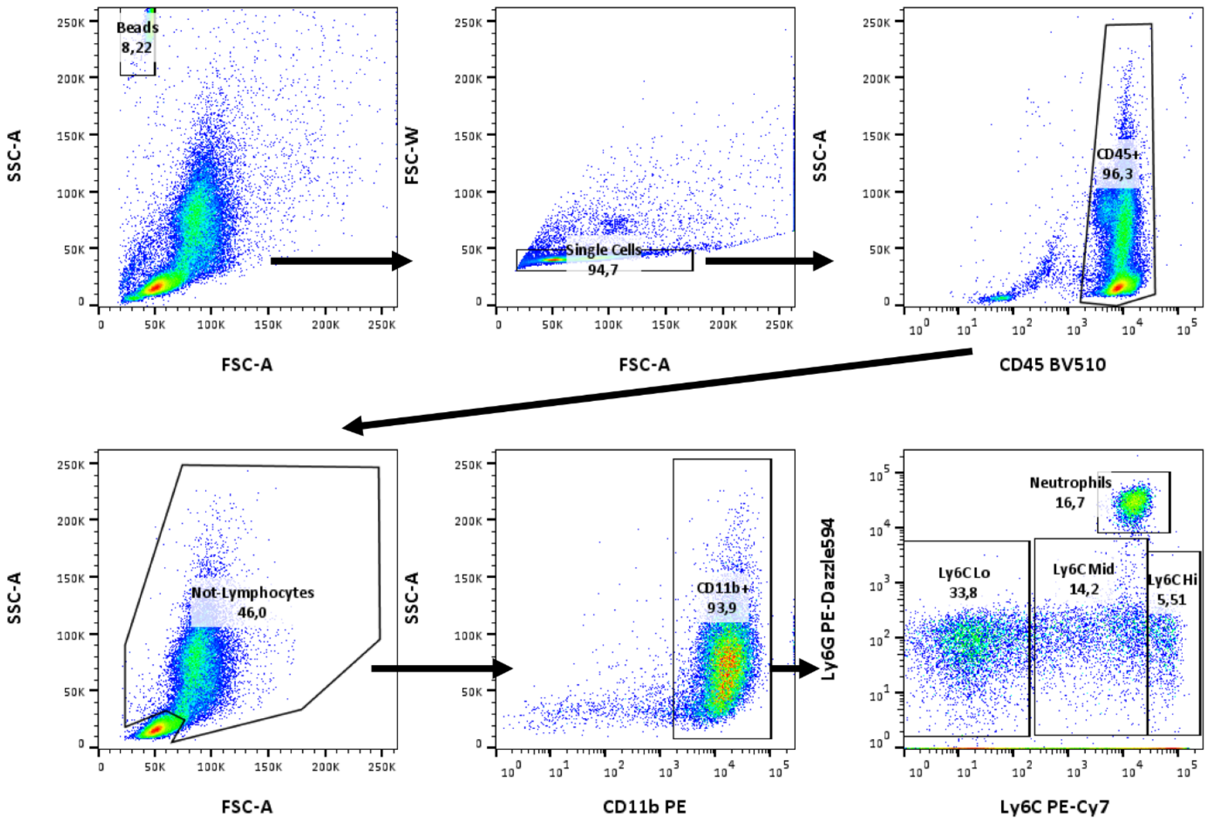

Supplemental Figure 1. Gating strategy for monocytes and neutrophils.

123Count Ebeads and doublet cells were excluded from analysis. Total lymphocytes were gated for CD45+ expression. Total monocytes and granulocytes were gated based on FSC and SSC profile and CD11b+ expression. Neutrophils were gated as Ly6G+ Ly6C+ cells. Monocytes were Ly6G- populations gated as Ly6C^{hi}, Ly6C^{mid}, or Ly6C^{lo} populations.

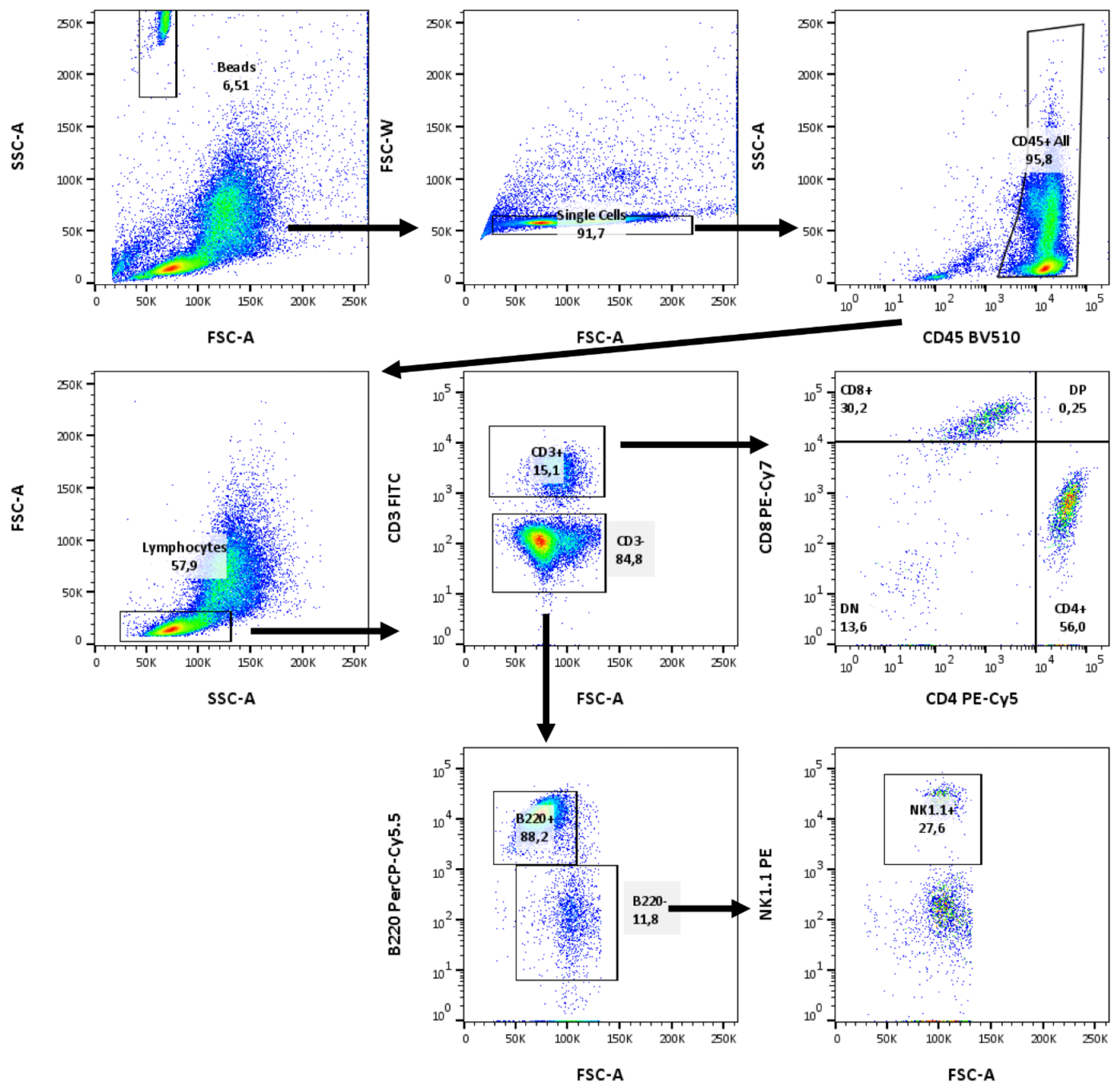

Supplemental Figure 2. Gating strategy for T cells, B cells, and NK cells.

123Count Ebeads and doublet cells were excluded from analysis. Total lymphocytes were gated for CD45+ expression. Total lymphocytes were gated based on FSC and SSC profile. T cells were gated as CD3+ expressing cells and further subdivided into CD8+ or CD4+ populations. B cells were gated as CD3- cells expressing B220. NK cells were gated as CD3-B220-NK1.1+ populations.

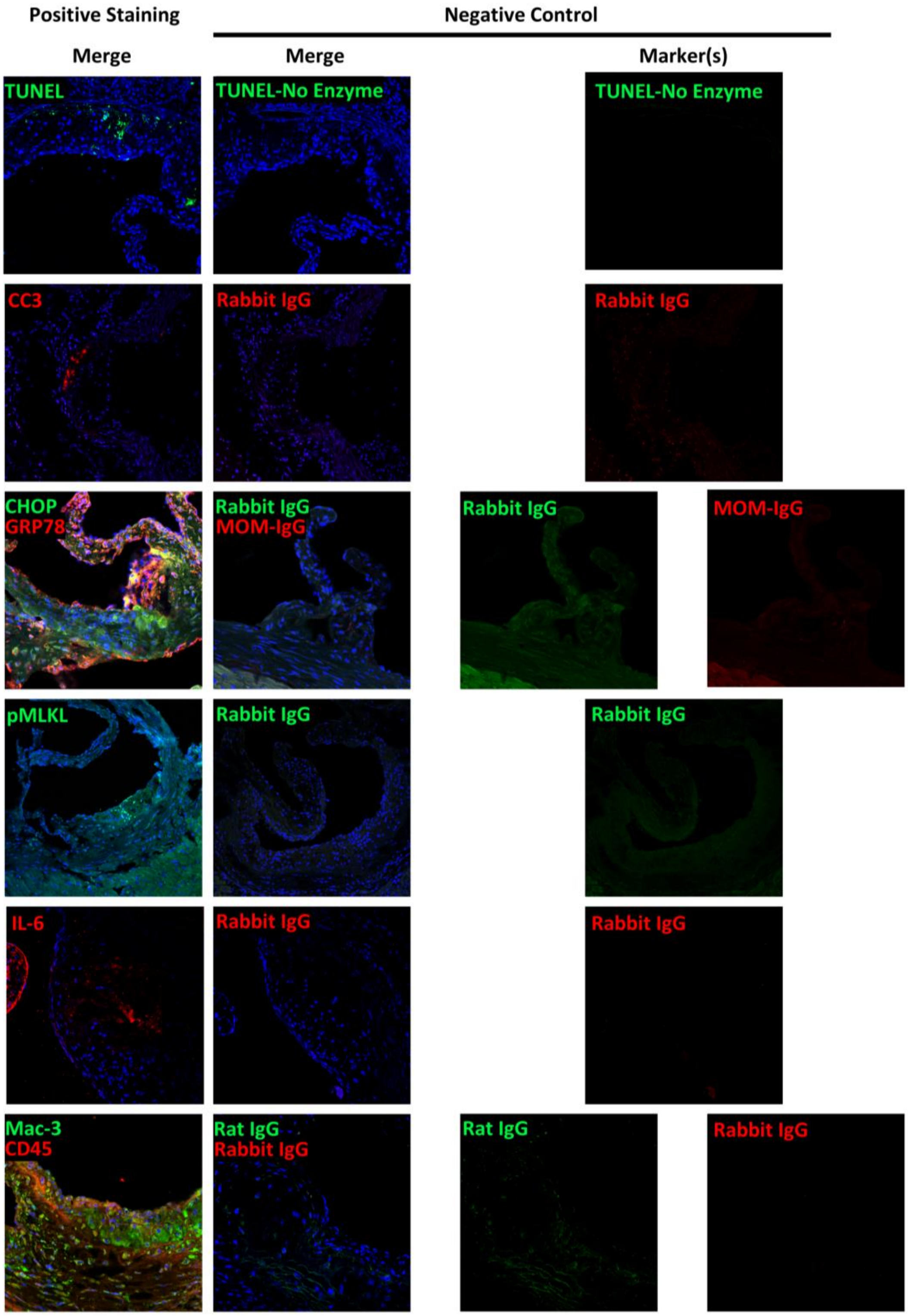

Supplemental Figure 3. Negative controls for immunofluorescence staining in atherosclerotic sections

Immunofluorescent staining for corresponding markers in atherosclerotic plaque sections was conducted as outlined in the methods. Negative controls for each corresponding marker was conducted using no enzyme control (for TUNEL), rabbit isotype control IgG (for CC3, CHOP, pMLKL, IL-6 and CD45), Mouse-on-Mouse (MOM) IgG (for GRP78), or rat isotype control IgG (for Mac-3) with their corresponding secondary antibodies or fluorescent probes as outlined in the methods.

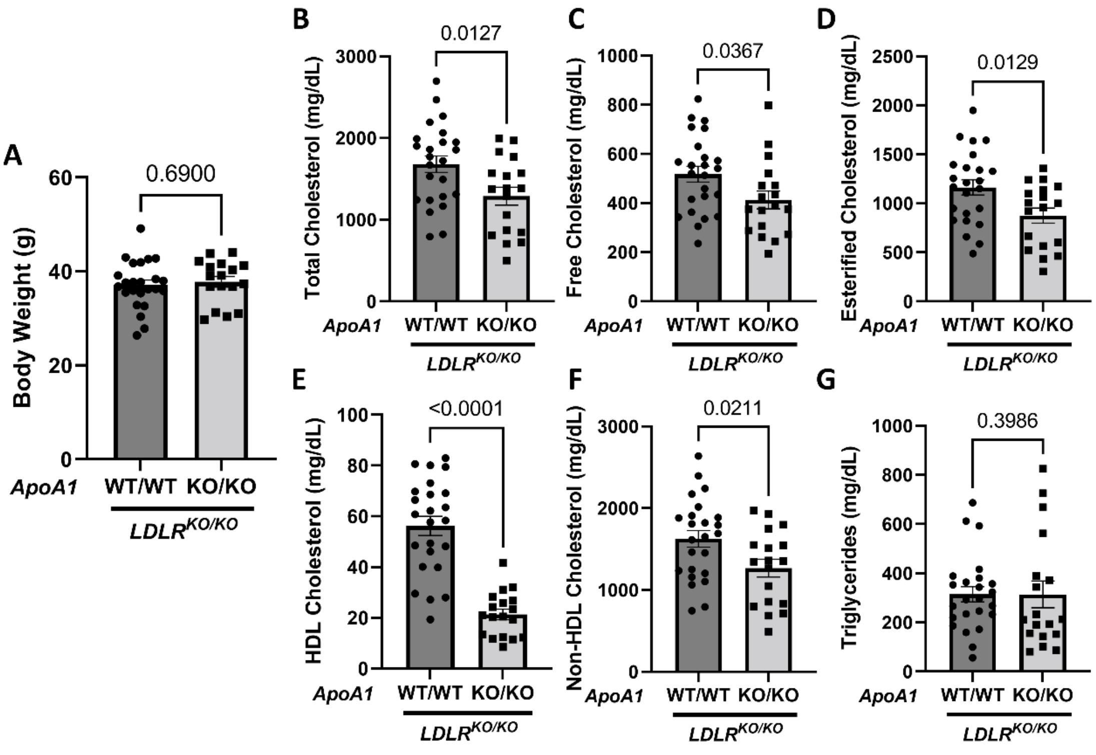

Supplemental Figure 4. Body weight and plasma lipid concentrations of ApoA1$^{WT/WT}$LDLR$^{KO/KO}$ mice and ApoA1$^{KO/KO}$LDLR$^{KO/KO}$ mice.

Male ApoA1$^{WT/WT}$LDLR$^{KO/KO}$ (LDLR$^{KO/KO}$) and ApoA1$^{KO/KO}$LDLR$^{KO/KO}$ mice (n=22, 20) were fed a high-fat diet for 10 weeks. **A**. Body weights measured at the end of feeding period. Plasma samples were isolated from blood and assessed using enzymatic colorimetric assays for **B**, total cholesterol; **C**, free cholesterol; **D**, esterified cholesterol; **E**, HDL cholesterol; **F**, non-HDL cholesterol; and **E**, triglycerides. For **A – F,** statistical analysis was conducted using unpaired t test. For **G,** statistical analysis was conducted using Mann Whitney test. Statistical significance is considered when $p<0.05$. Data represents mean ± SEM.

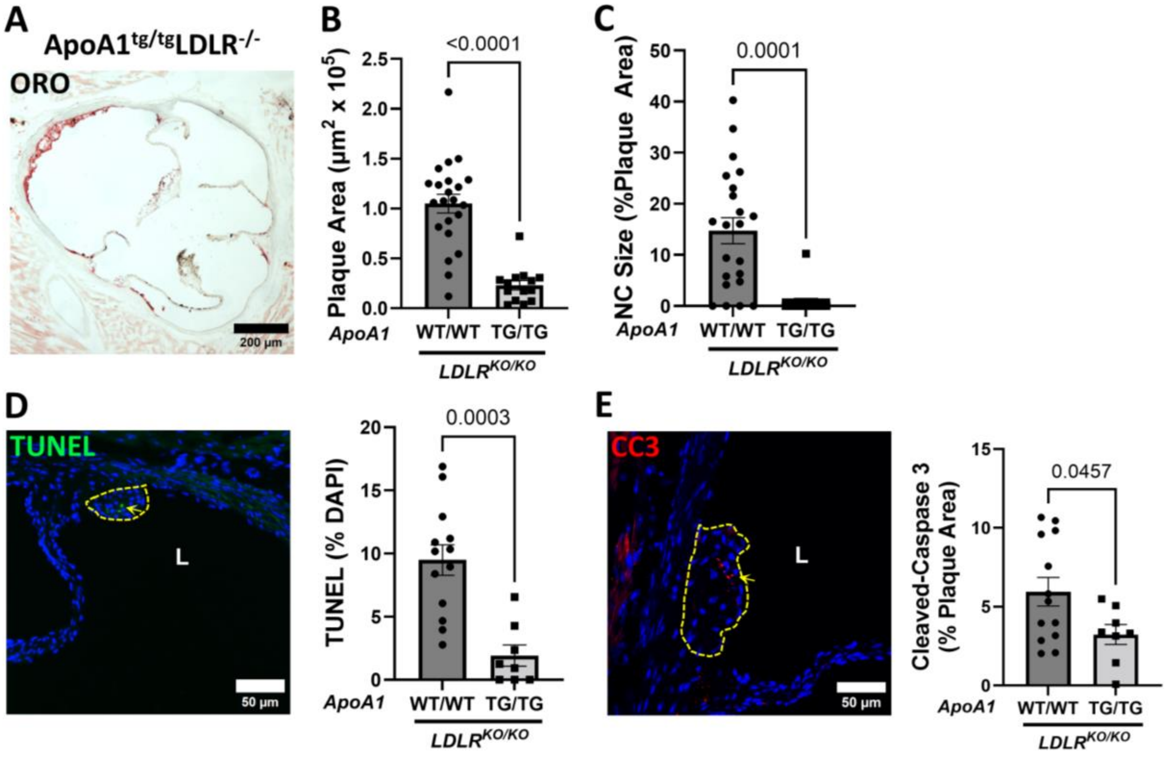

Supplemental Figure 5. Effect of transgenic (TG) overexpression of ApoA1 in LDLR$^{KO/KO}$ mice on atherosclerotic plaque and necrotic core sizes and levels of plaque apoptosis.

Male 10-week-old ApoA1$^{TG/TG}$LDLR$^{KO/KO}$ were fed a high-fat diet for 10 weeks (n=14). Results are compared to ApoA1$^{WT/WT}$LDLR$^{KO/KO}$ mice described in Figure 1. **A,** Representative images of oil-red-O (ORO) stained atherosclerotic plaques from aortic sinus cross-sections. **B,** Quantification of peak plaque area taken at the apex of aortic sinus plaque profiles. **C,** Quantification of necrotic core area relative to peak plaque area. **D,** Representative image and quantification of TUNEL-positive nuclei within the atherosclerotic plaques (n=8). **E**, Representative image and quantification of CC3-positive area relative to the lesional area (n=8). Yellow dashed line outlines the plaque area. "L" represents the lumen of the aortic valve leaflet. For **B and C,** statistical analysis was conducted using Mann Whitney test. For **D and E,** statistical analysis was conducted using unpaired t test. Statistical significance is considered when $p<0.05$. Data represents mean ± SEM.

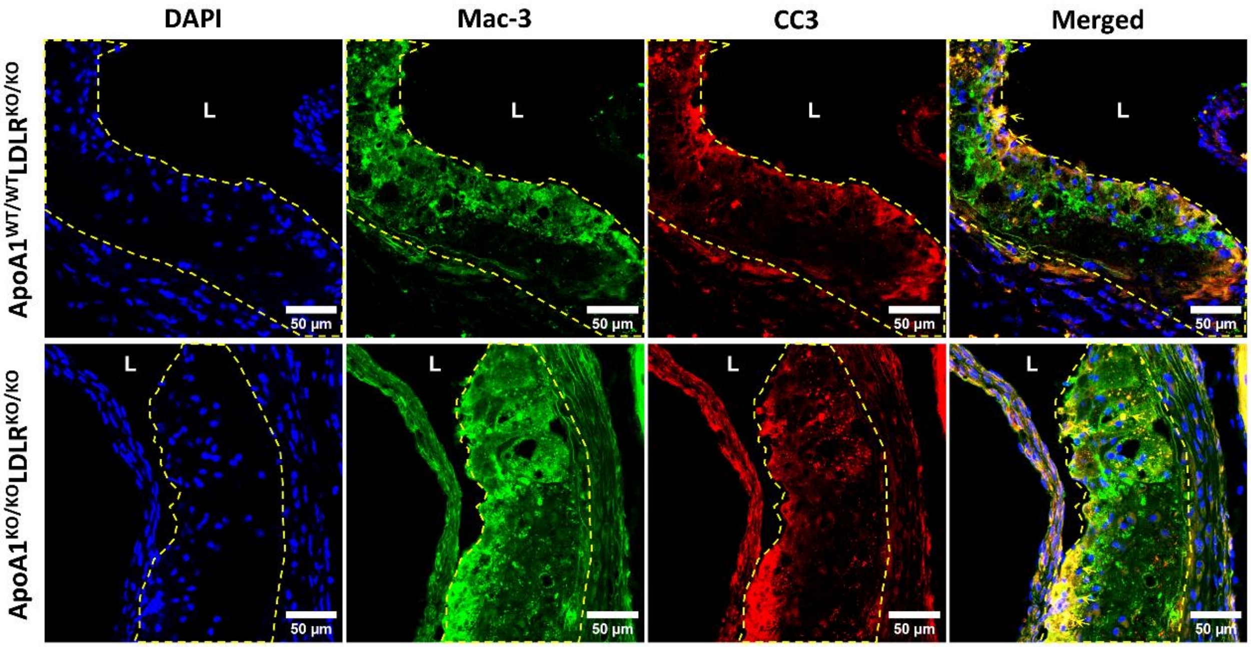

Supplemental Figure 6. Cleaved-caspase 3 immunofluorescence staining co-localizes with macrophages in atherosclerotic plaques.

Representative atherosclerotic plaque sections from ApoA1$^{WT/WT}$LDLR$^{KO/KO}$ (LDLR$^{KO/KO}$) and ApoA1$^{KO/KO}$LDLR$^{KO/KO}$ mice were probed with antibodies for Mac-3 (green) and CC3 (red) and counter-stained with DAPI (blue). Yellow dashed line outlines the plaque area. “L” represents the lumen of the aortic valve leaflet.

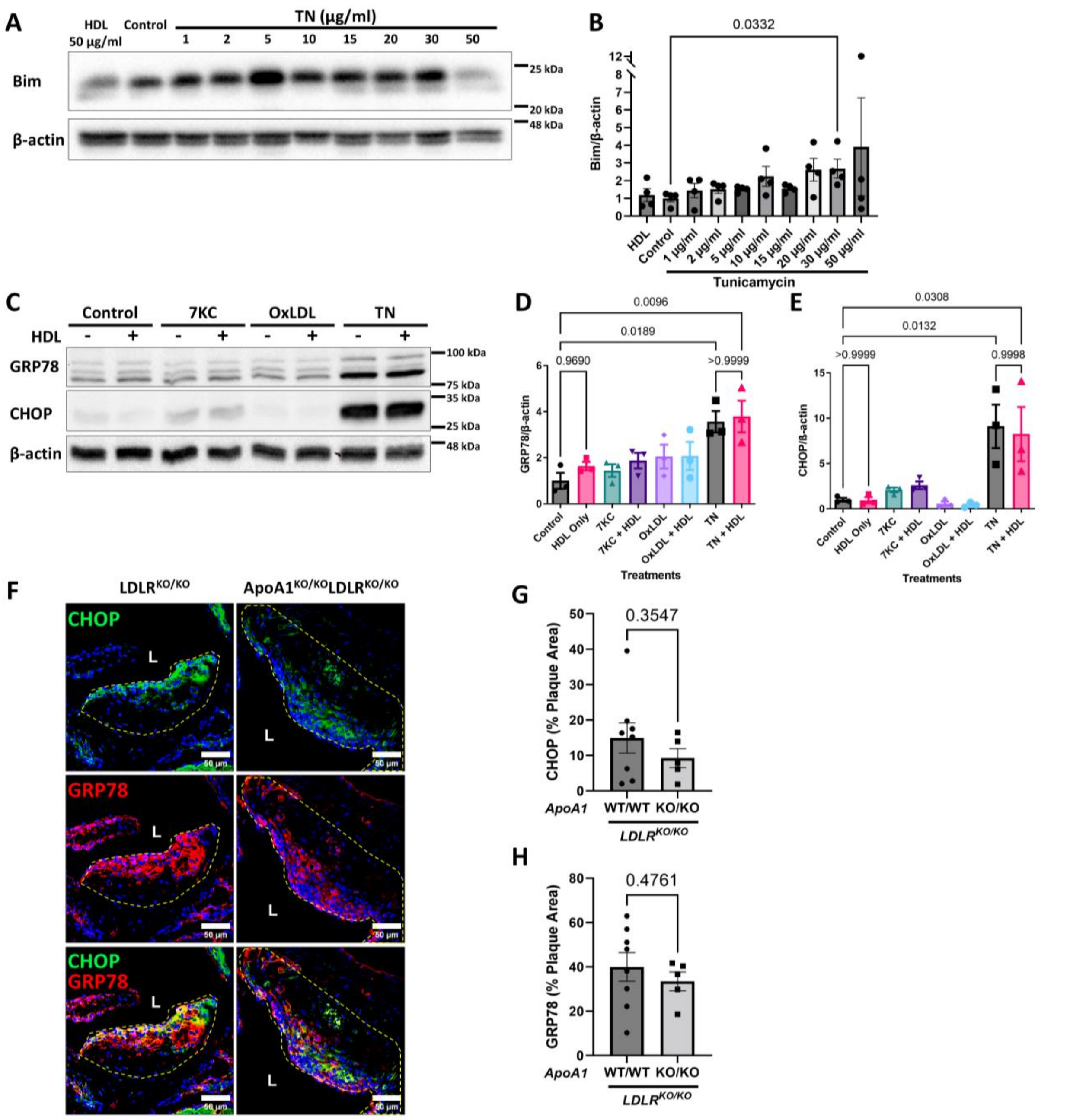

Supplemental Figure 7. HDL or ApoA1 does not change protein expression of GRP78 and CHOP in peritoneal macrophages and atherosclerotic plaques.

Mouse peritoneal macrophages (MPMs) from wild-type mice were isolated following thioglycolate induced peritonitis. MPMs were stressed with increasing concentrations of tunicamycin (TN) for 8 hours in newborn calf lipoprotein deficient serum. **A,** Representative blot for Bim expression. β-actin used as loading control. **B**, Relative fold change in Bim expression normalized to control (n=4). **C,** MPMs were stressed with 10

ug/ml 7-ketocholesterol (7KC), 100 ug/ml oxidized LDL (OxLDL), or 10 ug/ml TN with or without 50 ug/ml human high-density lipoprotein (HDL) for 24 hours in lipoprotein deficient serum. Representative blots for GRP78 and CHOP protein expression. β-actin used as loading control. **D,** Relative fold change in GRP78 and **E.** CHOP protein expression normalized to control (n=3). **F,** Representative images of GRP78 and CHOP immunofluorescent staining in aortic sinus atherosclerotic plaques of male $ApoA1^{WT/WT}LDLR^{KO/KO}$ and $ApoA1^{KO/KO}LDLR^{KO/KO}$ mice fed a high-fat diet for 10 weeks. Yellow dashed line outlines the plaque area. "L" represents the lumen of the aortic valve leaflet. **G,** quantification of CHOP and **H,** GRP78 staining relative to plaque area (n=8, 5). For **B, D and E,** statistical analysis was conducted using one-way ANOVA with Tukey post-hoc multiple comparisons test. For **G and H,** statistical analysis was conducted using an unpaired t test. Statistical significance is considered when $p<0.05$. Data represents mean ± SEM.

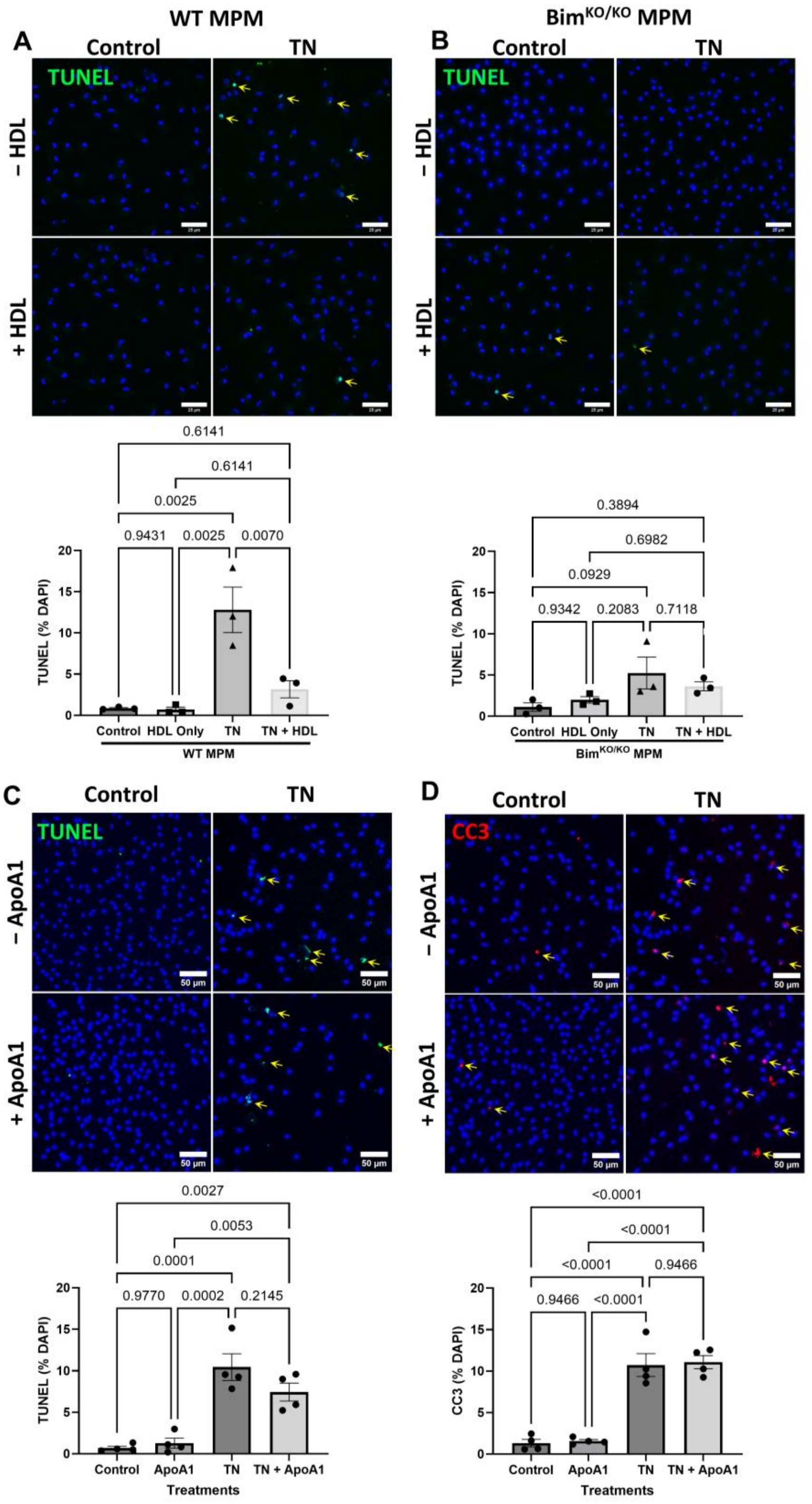

WT MPM
A
Control
TN
TUNEL
– HDL
+ HDL
Bim$^{KO/KO}$ MPM
B
Control
TN
TUNEL
– HDL
+ HDL
0.6141
0.6141
0.0025
0.9431
0.0025
0.0070
TUNEL (% DAPI)
Control
HDL Only
TN
TN + HDL
WT MPM
0.3894
0.6982
0.0929
0.9342
0.2083
0.7118
TUNEL (% DAPI)
Control
HDL Only
TN
TN + HDL
Bim$^{KO/KO}$ MPM
C
Control
TN
TUNEL
– ApoA1
+ ApoA1
50 µm
D
Control
TN
CC3
– ApoA1
+ ApoA1
50 µm
0.0027
0.0053
0.0001
0.9770
0.0002
0.2145
TUNEL (% DAPI)
Control
ApoA1
TN
TN + ApoA1
Treatments
<0.0001
<0.0001
<0.0001
0.9466
0.9466
<0.0001
CC3 (% DAPI)
Control
ApoA1
TN
TN + ApoA1
Treatments

Supplemental Figure 8. HDL but not ApoA1 treatment protects WT peritoneal macrophages from tunicamycin induced cell death but does not offer additional protection in apoptosis resistant Bim$^{KO/KO}$ macrophages.

Thioglycolate-elicited mouse peritoneal macrophages (MPM) from wild-type C57/BL6 (WT) and Bim$^{KO/KO}$ mice were harvested and cultured in media containing lipoprotein deficient serum and treated with or without 50 μg/ml human high-density lipoprotein (HDL) and with or without 10 μg/ml tunicamycin (TN) for 24 hours. **A,** Representative images and quantification of TUNEL (green) positive nuclei in MPMs from WT mice exposed to HDL, TN, or both. Nuclei stained blue with DAPI. **B,** Representative images and quantification of TUNEL (green) positive nuclei in MPMs from Bim$^{KO/KO}$ mice exposed to HDL, TN, or both. MPMs from WT mice were harvested and cultured in media containing lipoprotein deficient serum and treated with or without 50 μg/ml human ApoA1 and with or without 10 μg/ml TN for 24 hours. **C**, Representative images and quantification of TUNEL (green) positive nuclei in MPMs from WT mice exposed to ApoA1, TN, or both. **D**, Representative images and quantification of CC3 (red) stained MPMs from WT mice exposed to ApoA1, TN, or both. For **A – D**, statistical analysis was conducted using one-way ANOVA with Tukey post-hoc multiple comparisons test. Statistical significance is considered when $p<0.05$. Data represents mean ± SEM.

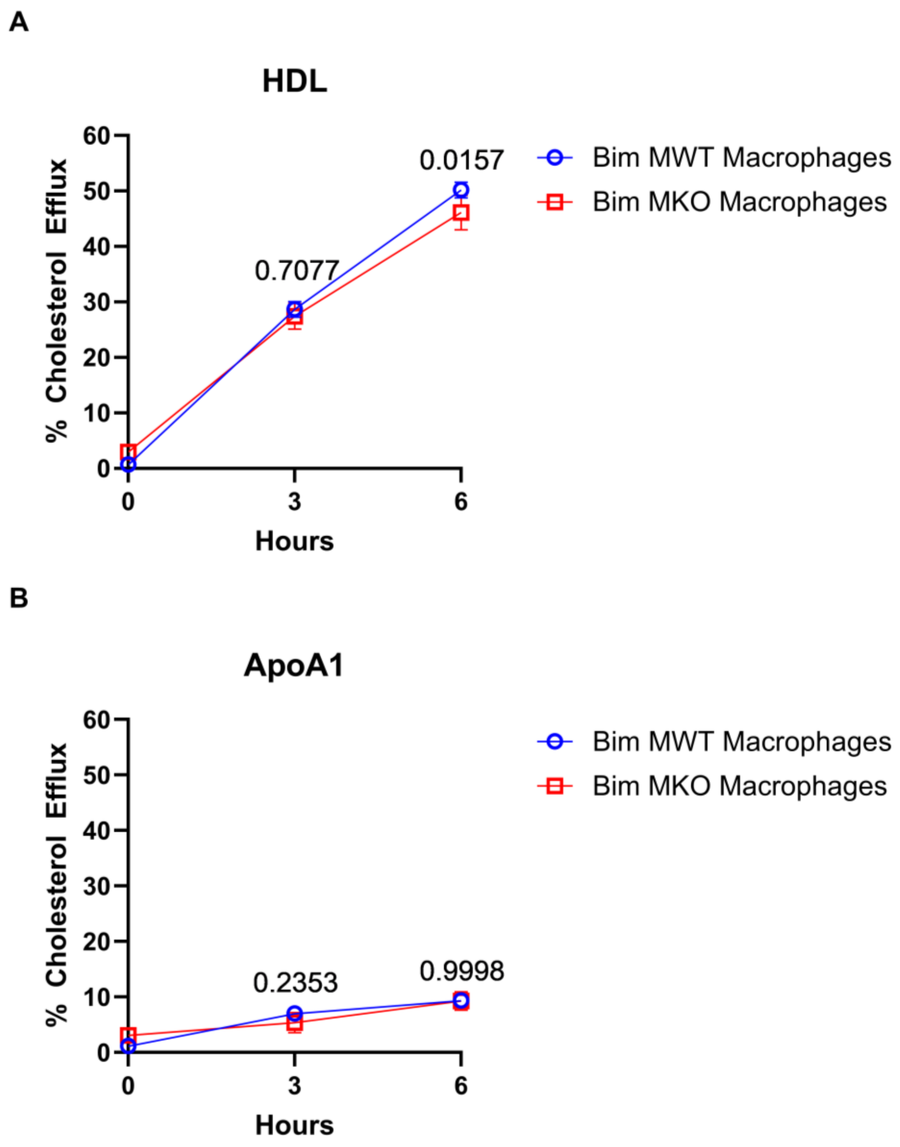

Supplemental Figure 9. Cholesterol Efflux to HDL and ApoA1 in Bim^{MWT} and Bim^{MKO} peritoneal macrophages.

Thioglycolate-elicited mouse peritoneal macrophages (MPM) from Bim^{MWT} and Bim^{MKO} mice were harvested and exposed to either human HDL or human ApoA1. Cholesterol efflux was measured at 0 hours, 3 hours, and 6 hours following exposure to HDL or ApoA1. Statistical analysis was conducted using two-way ANOVA with Šídák's multiple comparisons test. Statistical significance is considered when $p<0.05$. Data represents mean ± SD.

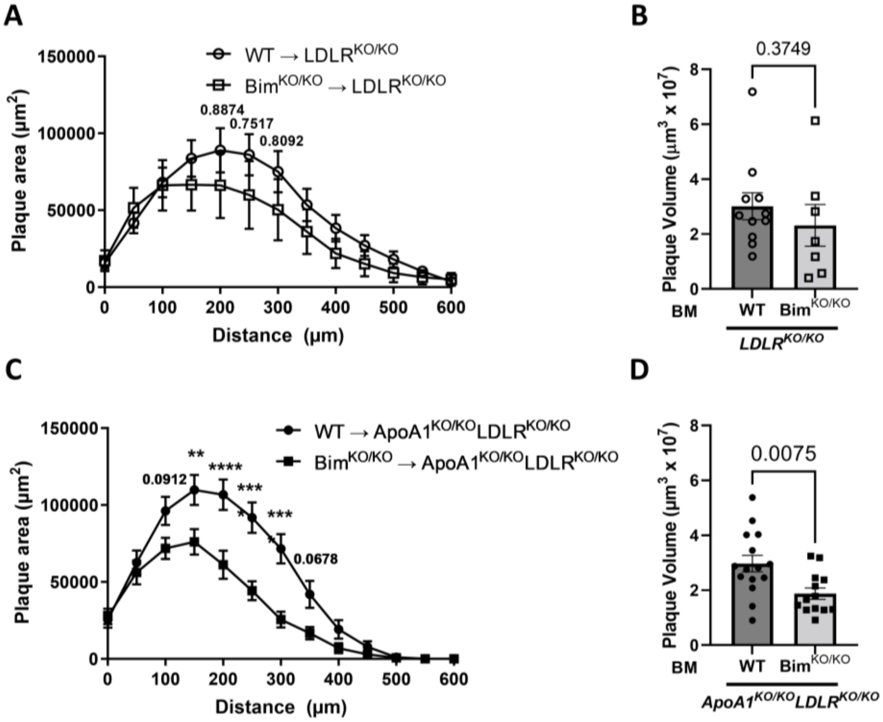

Supplemental Figure 10. Atherosclerotic plaque profiles and volumes for LDLR$^{KO/KO}$ and ApoA1$^{KO/KO}$LDLR$^{KO/KO}$ mice transplanted with WT or Bim$^{KO/KO}$ bone marrow fed a high-fat diet for 10 weeks.

A, Profile of atherosclerotic plaque area of transplanted LDLR$^{KO/KO}$ mice in 50 µm cross sections from the beginning of the aortic annulus. **B,** Plaque volumes of bone marrow transplanted LDLR$^{KO/KO}$ mice. **C,** Profile of atherosclerotic plaque area of transplanted ApoA1$^{KO/KO}$LDLR$^{KO/KO}$ mice in 50 µm cross sections from the beginning of the aortic annulus. **D,** Plaque volumes of bone marrow transplanted ApoA1$^{KO/KO}$LDLR$^{KO/KO}$ mice. For **A and C**, statistical analysis was done using two-way ANOVA with Tukey post-hoc multiple comparisons test. For **B**, statistical analysis was done using Mann Whitney test. For **D**, statistical analysis was done using unpaired t test. ** represents p=0.0027 and **** represents p<0.0001. Statistical significance is considered when p<0.05. Data represents mean ± SEM.

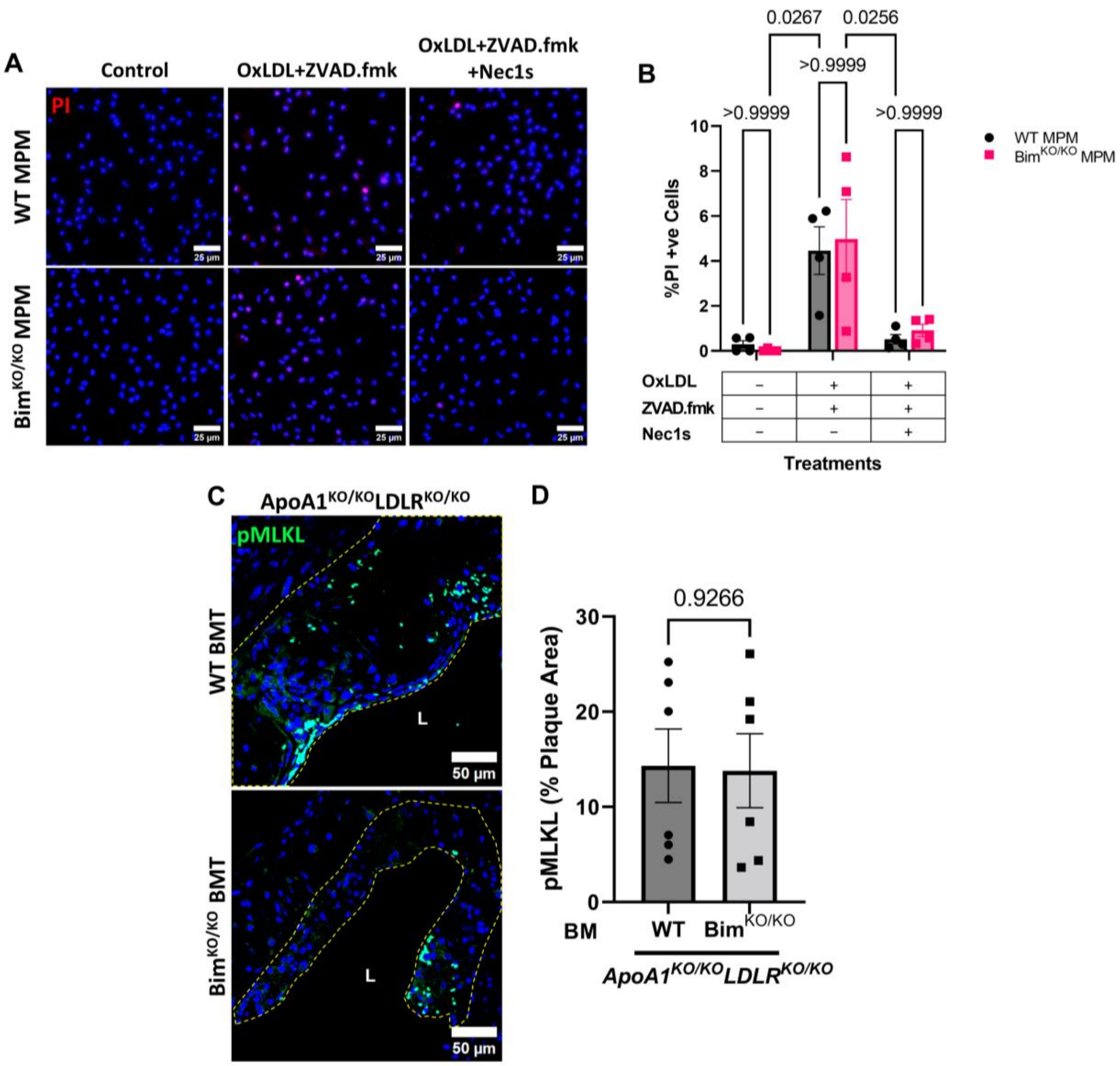

Supplemental Figure 11. Bim deficiency does not affect necroptosis in peritoneal macrophages and atherosclerosis plaques.

Necroptosis was induced in thioglycolate-elicited MPMs from WT and Bim$^{KO/KO}$ mice with 50 µg/ml oxidized LDL (OxLDL) and 50 µM carbobenzoxy-valyl-alanyl-aspartyl-[Omethyl]-fluoromethylketone (ZVAD.fmk) and with or without 50 µM necrostatin-1s (Nec1s). **A**. Representative images of propidium iodide (PI, red) staining in WT MPMs (top row) and Bim$^{KO/KO}$ MPMs (bottom row). Nuclei stained with DAPI (blue). **B**. Quantification of PI positive cells in MPMs from WT and Bim$^{KO/KO}$ mice (n=4, 4). **C**. Representative images of phosphorylated mixed lineage kinase domain like pseudokinase (pMLKL)

immunofluorescent staining in aortic sinus atherosclerotic plaques of male $ApoA1^{KO/KO}LDLR^{KO/KO}$ mice transplanted with WT (top) or $Bim^{KO/KO}$ (bottom) bone marrow fed a high-fat diet for 10 weeks. Yellow dashed line outlines the plaque area. “L” represents the lumen of the aortic valve leaflet. **D**. Quantification of pMLKL staining relative to plaque area (n=6, 6). For **B**, Statistical analysis was conducted using one-way ANOVA with Tukey post-hoc multiple comparisons test. For **D**, Statistical analysis was conducted using unpaired t test. Statistical significance is considered when $p<0.05$. Data represents mean ± SEM.

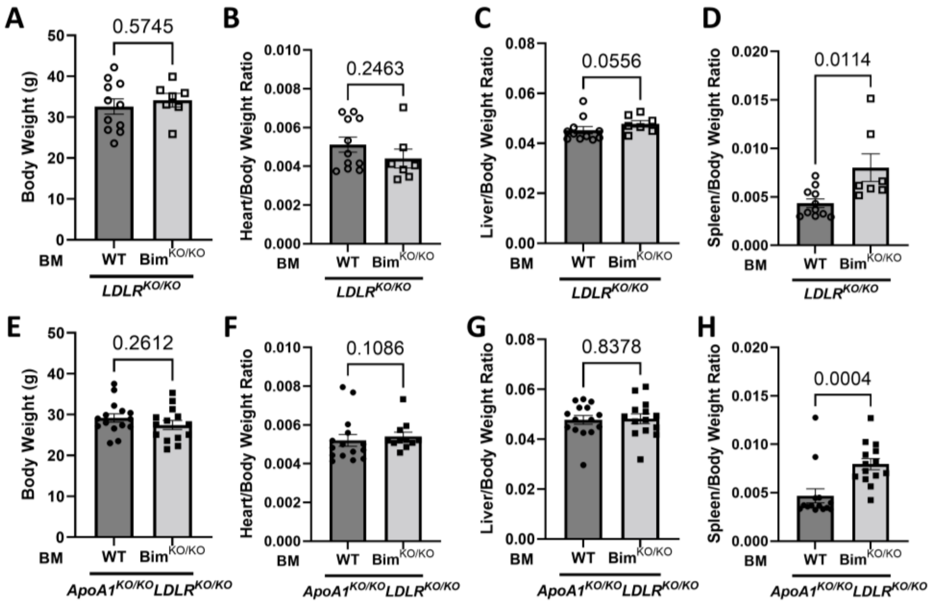

Supplemental Figure 12. Weights for LDLR$^{KO/KO}$ and ApoA1$^{KO/KO}$LDLR$^{KO/KO}$ mice transplanted with WT or Bim$^{KO/KO}$ bone marrow fed a high-fat diet for 10 weeks.

A. Body weight of bone marrow transplanted LDLR$^{KO/KO}$ mice at end of diet feeding (n=11, 7). **B,** Heart weight; **C**, liver weight; **D**, and spleen weight relative to body weight ratio. **E.** Body weight of bone marrow transplanted ApoA1$^{KO/KO}$LDLR$^{KO/KO}$ mice at end of diet feeding (n=15, 14). **F,** Heart weight; **G**, liver weight; and **H**, spleen weight relative to body weight ratio. For **A, E, and G**, statistical analysis was conducted using unpaired t test. For **B – D, F, and H**, Statistical analysis was done using Mann Whitney test. Statistical significance is considered when $p<0.05$. Data represents mean ± SEM.

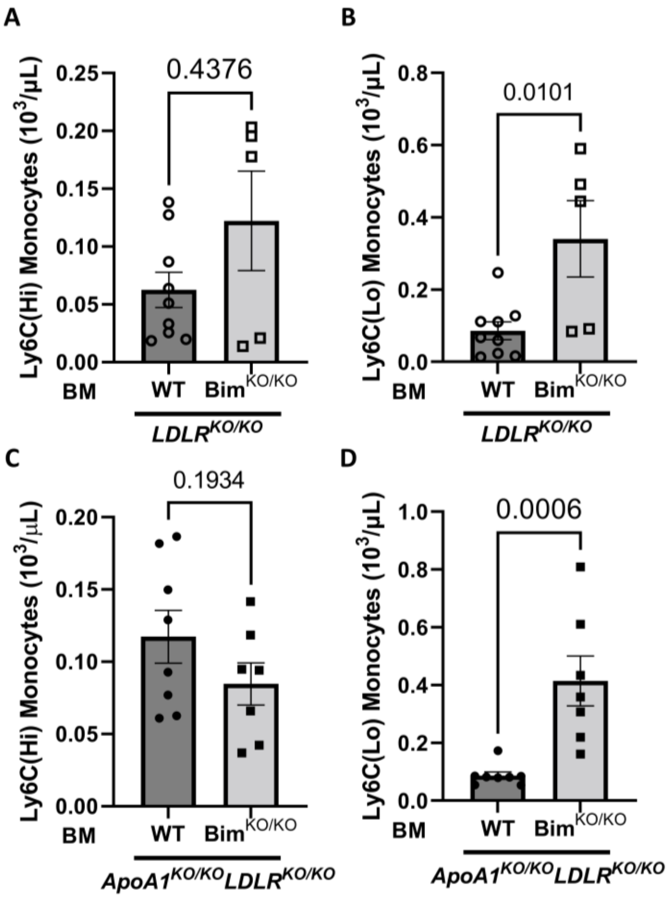

Supplemental Figure 13. Circulating monocyte subsets in blood from LDLR$^{KO/KO}$ and ApoA1$^{KO/KO}$LDLR$^{KO/KO}$ mice transplanted with WT or Bim$^{KO/KO}$ bone marrow fed a high-fat diet for 10 weeks.

Peripheral blood was collected by cardiac puncture from 10-week HFD fed LDLR$^{KO/KO}$ and ApoA1$^{KO/KO}$LDLR$^{KO/KO}$ mice transplanted with WT or Bim$^{KO/KO}$ BM. Monocyte concentrations from peripheral blood samples of mice were analyzed through flow cytometry. Absolute concentrations of **A**, Ly6C^{hi}; and **B**, Ly6C^{lo} CD11b+Ly6G- monocytes in blood from LDLR$^{KO/KO}$ BMT mice (n=9, 5). Absolute concentrations of **C**, Ly6C^{hi}; and **D**, Ly6C^{lo} CD11b+Ly6G- monocytes in blood from ApoA1$^{KO/KO}$LDLR$^{KO/KO}$ BMT mice (n=8, 7). For **B and C**, statistical analysis was conducted using unpaired t test. For **A and D**, statistical analysis was done using Mann Whitney test. Statistical significance is considered when $p<0.05$. Data represents mean ± SEM.

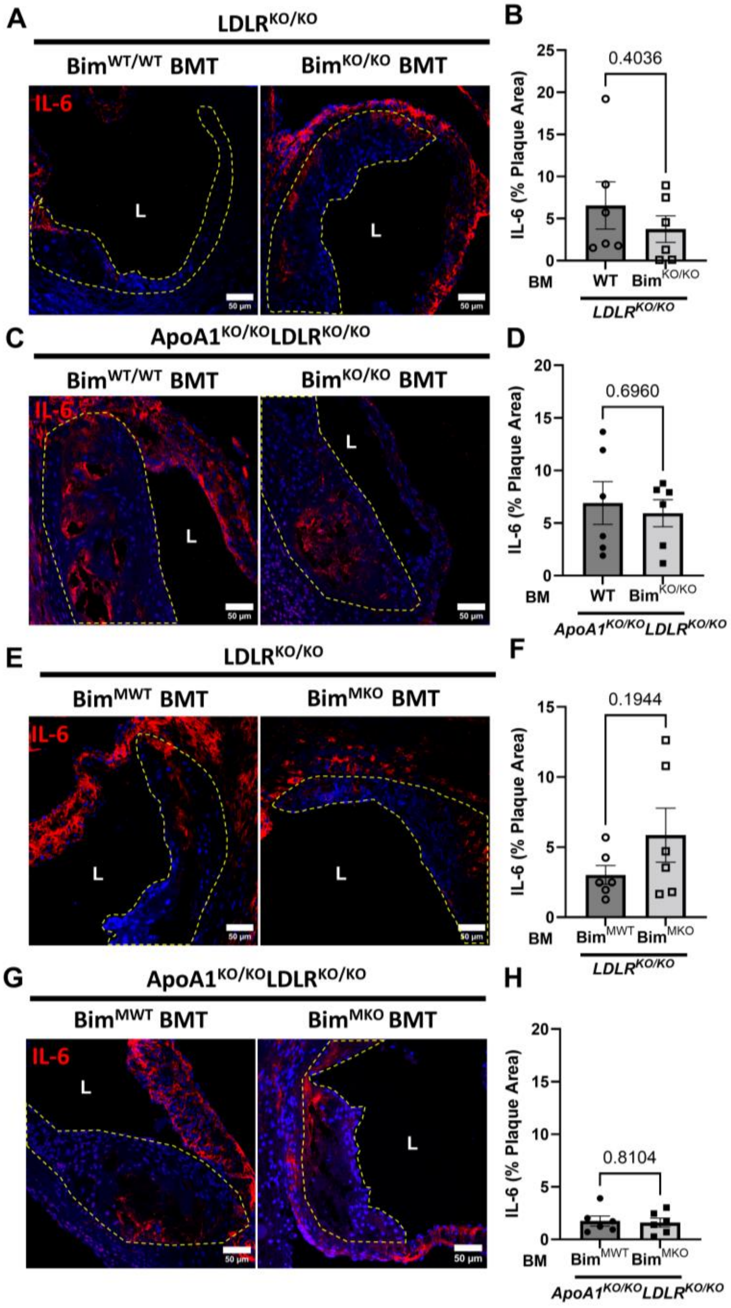
A
LDLR^KO/KO
Bim^WT/WT BMT
Bim^KO/KO BMT
IL-6
L
50 μm
B
IL-6 (% Plaque Area)
0.4036
BM
WT
Bim^KO/KO
LDLR^KO/KO
C
ApoA1^KO/KO LDLR^KO/KO
Bim^WT/WT BMT
Bim^KO/KO BMT
D
0.6960
ApoA1^KO/KO LDLR^KO/KO
E
LDLR^KO/KO
Bim^MWT BMT
Bim^MKO BMT
F
0.1944
Bim^MWT
Bim^MKO
G
ApoA1^KO/KO LDLR^KO/KO
H
0.8104

Supplemental Figure 14. IL-6 staining in atherosclerotic plaques from LDLR$^{KO/KO}$ and ApoA1$^{KO/KO}$LDLR$^{KO/KO}$ mice transplanted with WT, Bim$^{KO/KO}$, Bim^{MWT}, or Bim^{MKO} bone marrow fed a high-fat diet for 10 weeks.

A, Representative images and **B**, quantification of IL-6 (red) immunofluorescent staining as a percentage of aortic sinus atherosclerotic plaque area of LDLR$^{KO/KO}$ mice transplanted with WT or Bim$^{KO/KO}$ BM. **C**, Representative images and **D**, quantification of IL-6 staining as a percentage of aortic sinus atherosclerotic plaque area of ApoA1$^{KO/KO}$LDLR$^{KO/KO}$ mice transplanted with WT or Bim$^{KO/KO}$ BM. **E**, Representative images and **F**, quantification of IL-6 staining as a percentage of aortic sinus atherosclerotic plaque area of LDLR$^{KO/KO}$ mice transplanted with Bim^{MWT} or Bim^{MKO} BM. **G**, Representative images and **H**, quantification of IL-6 staining as a percentage of aortic sinus atherosclerotic plaque area of ApoA1$^{KO/KO}$LDLR$^{KO/KO}$ mice transplanted with Bim^{MWT} or Bim^{MKO} BM. Yellow dashed line outlines the plaque area. "L" represents the lumen of the aortic valve leaflet. Statistical analysis was conducted using unpaired t test. Statistical significance is considered when $p<0.05$. Data represents mean ± SEM.

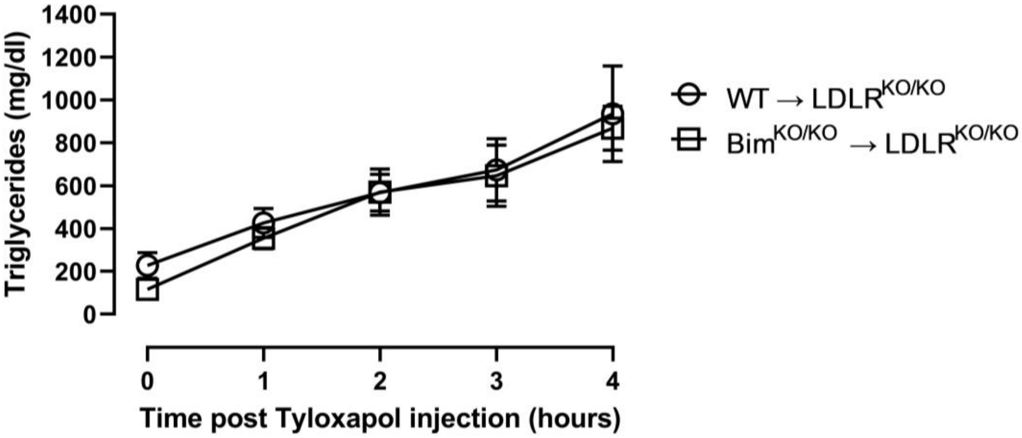

Supplemental Figure 15. Fasting triglyceride secretion is not altered by bone marrow Bim deficiency in 10 week high-fat diet fed LDLR$^{KO/KO}$ mice transplanted with WT or Bim$^{KO/KO}$ bone marrow.

Lipoprotein lipase was inhibited with intravenous injection of tyloxapol (500 mg/kg) following overnight fasting in 10 week high-fat diet fed LDLR$^{KO/KO}$ mice transplanted with WT or Bim$^{KO/KO}$ bone marrow. Blood was collected prior to and 1, 2, 3, and 4 hour(s) following tyloxapol injection and triglyceride concentration in plasma was measured (n=5, 4). Data represents mean ± SEM.

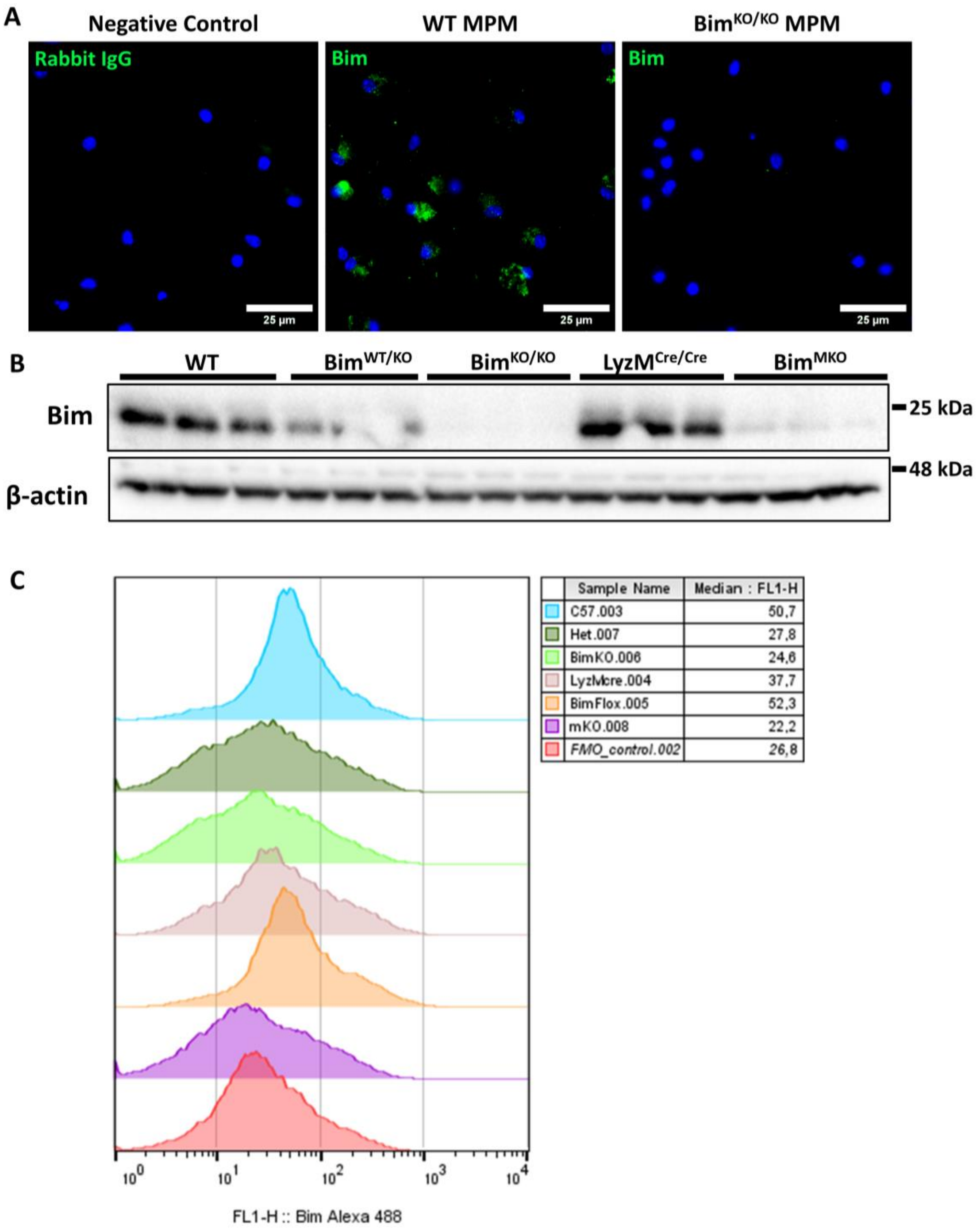

Supplemental Figure 16. Bim protein expression in Bim$^{KO/KO}$ and LysM$^{cre/cre}$Bim$^{fl/fl}$ macrophages.

Peritoneal macrophages were harvested from mice following thioglycolate elicited peritonitis. **A**, Representative images of MPMs from WT and Bim$^{KO/KO}$ mice stained for Bim protein (green). Nuclei stained blue with DAPI. **B**, MPMs from WT, Bim$^{WT/KO}$, Bim$^{KO/KO}$, LyzM$^{Cre/Cre}$, and Bim^{MKO} mice (n=3 for

each group) were lysed and probed for Bim protein by Western Blot. β-actin used as loading control. **C**, Analysis of Bim protein mean fluorescence intensity of CD11b+ F4/80+ MPMs from WT (C57), $Bim^{WT/KO}$ (Het), $Bim^{KO/KO}$ (BimKO), $LyzM^{Cre/Cre}$, $Bim^{fl/fl}$ (BimFlox), and Bim^{MKO} (mKO) mice run in flow cytometry.

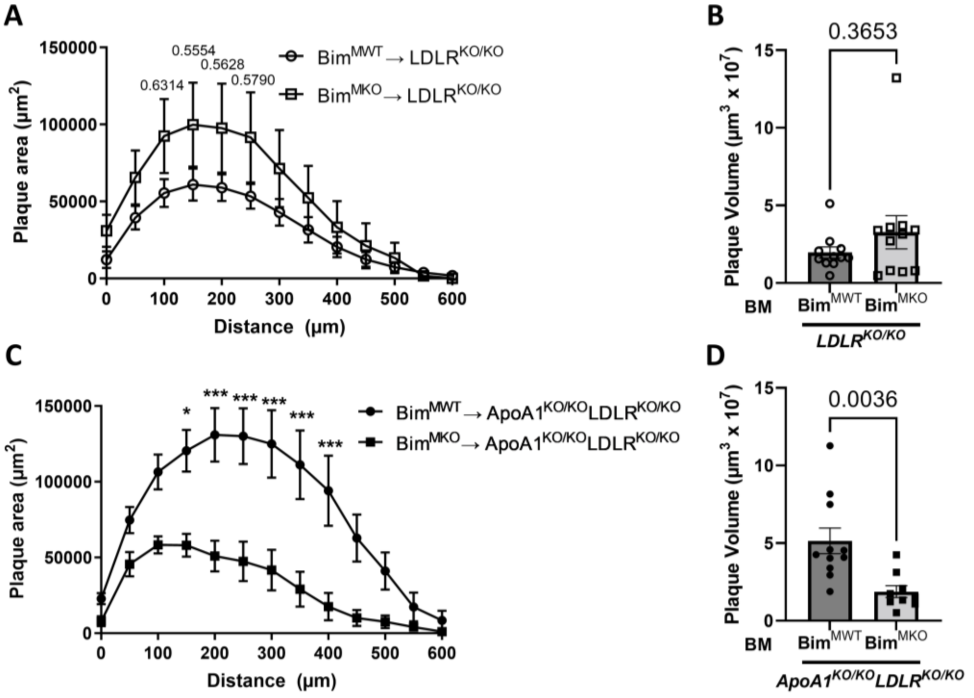

Supplemental Figure 17. Atherosclerotic plaque profiles and volumes of LDLR$^{KO/KO}$ and ApoA1$^{KO/KO}$LDLR$^{KO/KO}$ mice transplanted with Bim^{MWT} or Bim^{MKO} bone marrow fed a high-fat diet for 10 weeks.

A, Profile of atherosclerotic plaque area of transplanted LDLR$^{KO/KO}$ mice in 50 μm cross sections from the beginning of the aortic annulus. **B**, Plaque volumes of bone marrow transplanted LDLR$^{KO/KO}$ mice. **C**. Profile of atherosclerotic plaque area of transplanted ApoA1$^{KO/KO}$LDLR$^{KO/KO}$ mice in 50 μm cross sections from the beginning of the aortic annulus. **D**, Plaque volumes of bone marrow transplanted ApoA1$^{KO/KO}$LDLR$^{KO/KO}$ mice. For **A and C**, statistical analysis was done using two-way ANOVA with Tukey post-hoc multiple comparisons test. For **B,** statistical analysis was done using Mann Whitney test. **D,** Statistical analysis was done using unpaired t test. * represents p=0.0133, and *** represents p<0.001. Statistical significance is considered when p<0.05. Data represents mean ± SEM.

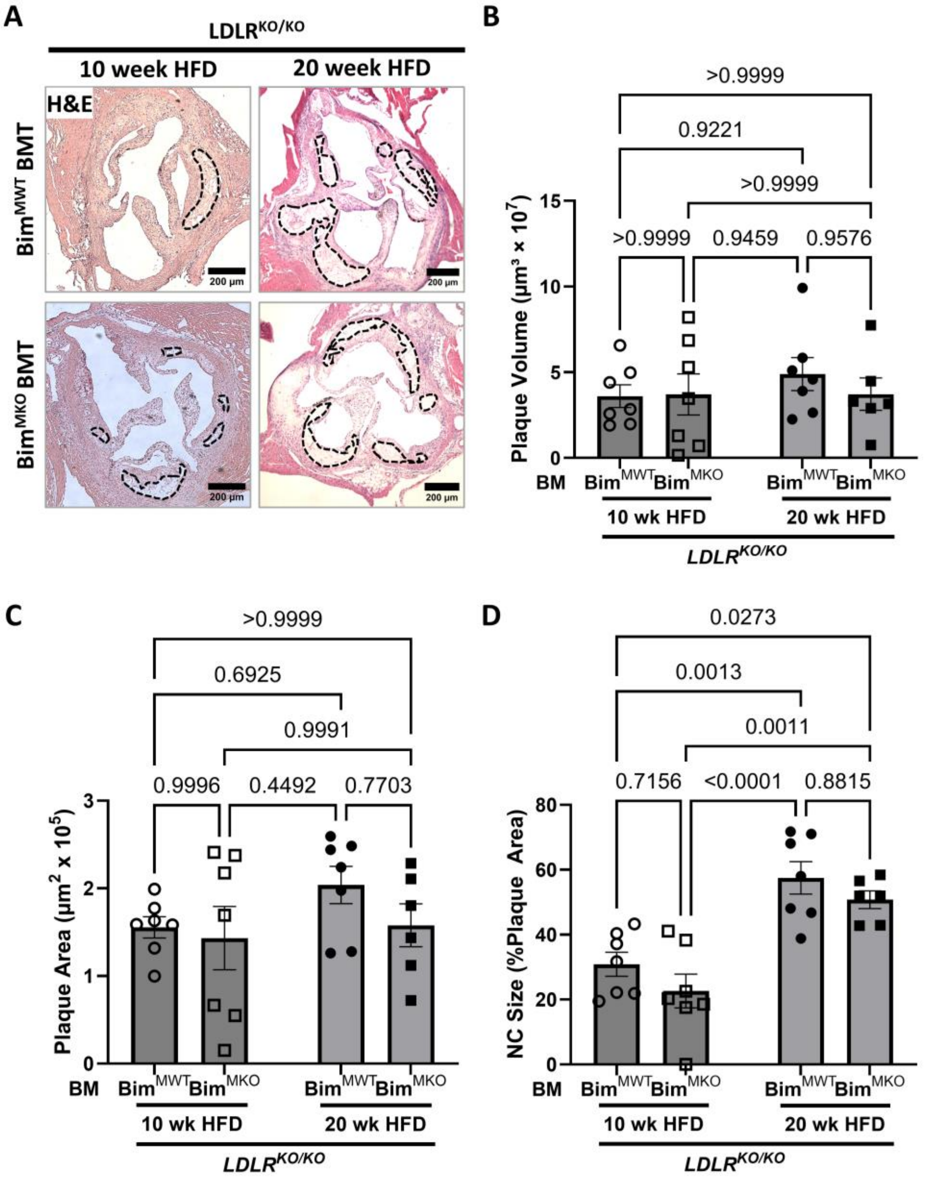

Supplemental Figure 18. Atherosclerosis analysis for female LDLR$^{KO/KO}$ mice transplanted with Bim^{MWT} or Bim^{MKO} bone marrow fed a high-fat diet for 10 or 20 weeks.

Female 10-week-old LDLR$^{KO/KO}$ were transplanted with bone marrow (BM) from LyzM$^{cre/cre}$ (Bim^{MWT}) or LyzM$^{cre/cre}$Bim$^{fl/fl}$ (Bim^{MKO}) donors and fed a high-fat diet (HFD) for 10 weeks (n=7, 7) or 20 weeks (n=7, 6). **A**, Representative images of H&E-stained atherosclerotic plaques from transplanted LDLR$^{KO/KO}$ mice. **B,** Plaque volumes; **C**, peak plaque area; and **D**, necrotic core size relative to peak plaque area of bone marrow transplanted LDLR$^{KO/KO}$ mice. Statistical analysis was conducted using two-way ANOVA with

Šídák's multiple comparisons test. Statistical significance is considered when $p<0.05$. Data represents mean ± SEM.

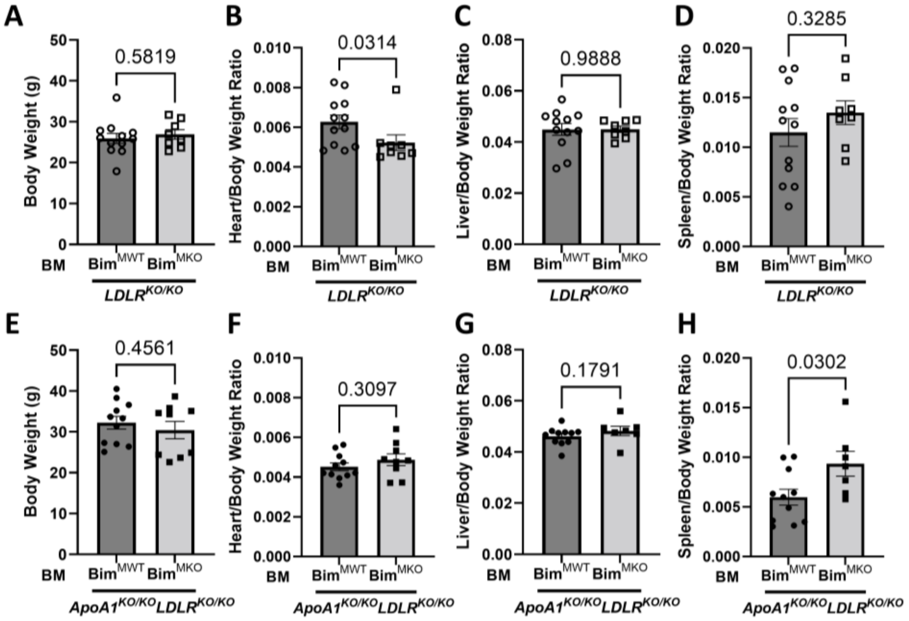

Supplemental Figure 19. Weights of LDLR$^{KO/KO}$ and ApoA1$^{KO/KO}$LDLR$^{KO/KO}$ mice transplanted with Bim^{MWT} or Bim^{MKO} bone marrow fed a high-fat diet for 10 weeks.

A. Body weight of bone marrow transplanted LDLR$^{KO/KO}$ mice at end of diet feeding (n=12, 8). **B,** Heart weight; **C**, liver weight; **D**, and spleen weight relative to body weight ratio. **E.** Body weight of bone marrow transplanted ApoA1$^{KO/KO}$LDLR$^{KO/KO}$ mice at end of diet feeding (n=11, 9). **F,** Heart weight; **G**, liver weight; and **H**, spleen weight relative to body weight ratio. **A, C, D, and F through H**, Statistical analysis was conducted using unpaired t test. **B and E**, Statistical analysis was conducted using Mann Whitney test. Statistical significance is considered when $p<0.05$. Data represents mean ± SEM.

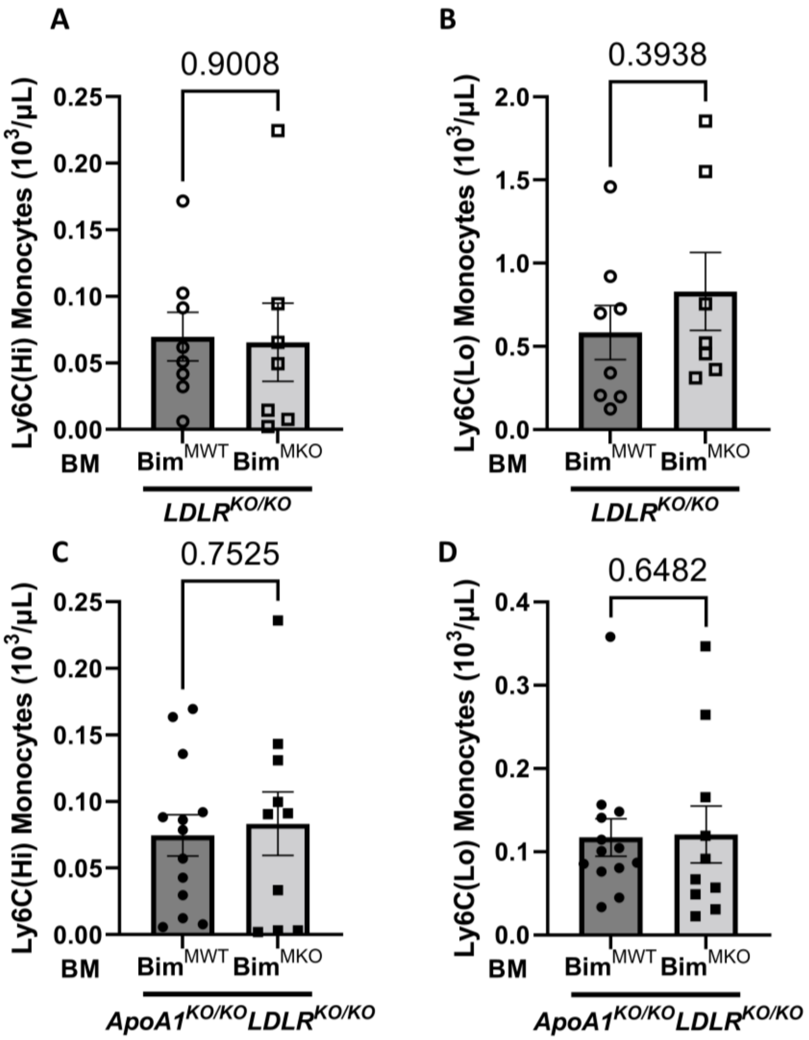

Supplemental Figure 20. Circulating monocyte subsets in blood isolated from LDLR$^{KO/KO}$ and ApoA1$^{KO/KO}$LDLR$^{KO/KO}$ mice transplanted with Bim^{MWT} or Bim^{MKO} bone marrow fed a high-fat diet for 10 weeks.

Peripheral blood was collected by cardiac puncture from 10-week HFD fed LDLR$^{KO/KO}$ and ApoA1$^{KO/KO}$LDLR$^{KO/KO}$ mice transplanted with Bim^{MWT} or Bim^{MKO} BM. Monocyte concentrations from peripheral blood samples of mice were analyzed through flow cytometry. Absolute concentrations of **A**, Ly6C^{hi} and **B**, Ly6C^{lo} CD11b+Ly6G- monocytes in blood from LDLR$^{KO/KO}$ BMT mice (n=8, 7). Absolute concentrations of **C**, Ly6C^{hi} and **D**, Ly6C^{lo} CD11b+Ly6G- monocytes in blood from ApoA1$^{KO/KO}$LDLR$^{KO/KO}$ BMT mice (n=13, 10). For **A - C**, statistical analysis was conducted using unpaired

t test. For **D**, statistical analysis was done using Mann Whitney test. Statistical significance is considered when $p<0.05$. Data represents mean ± SEM.

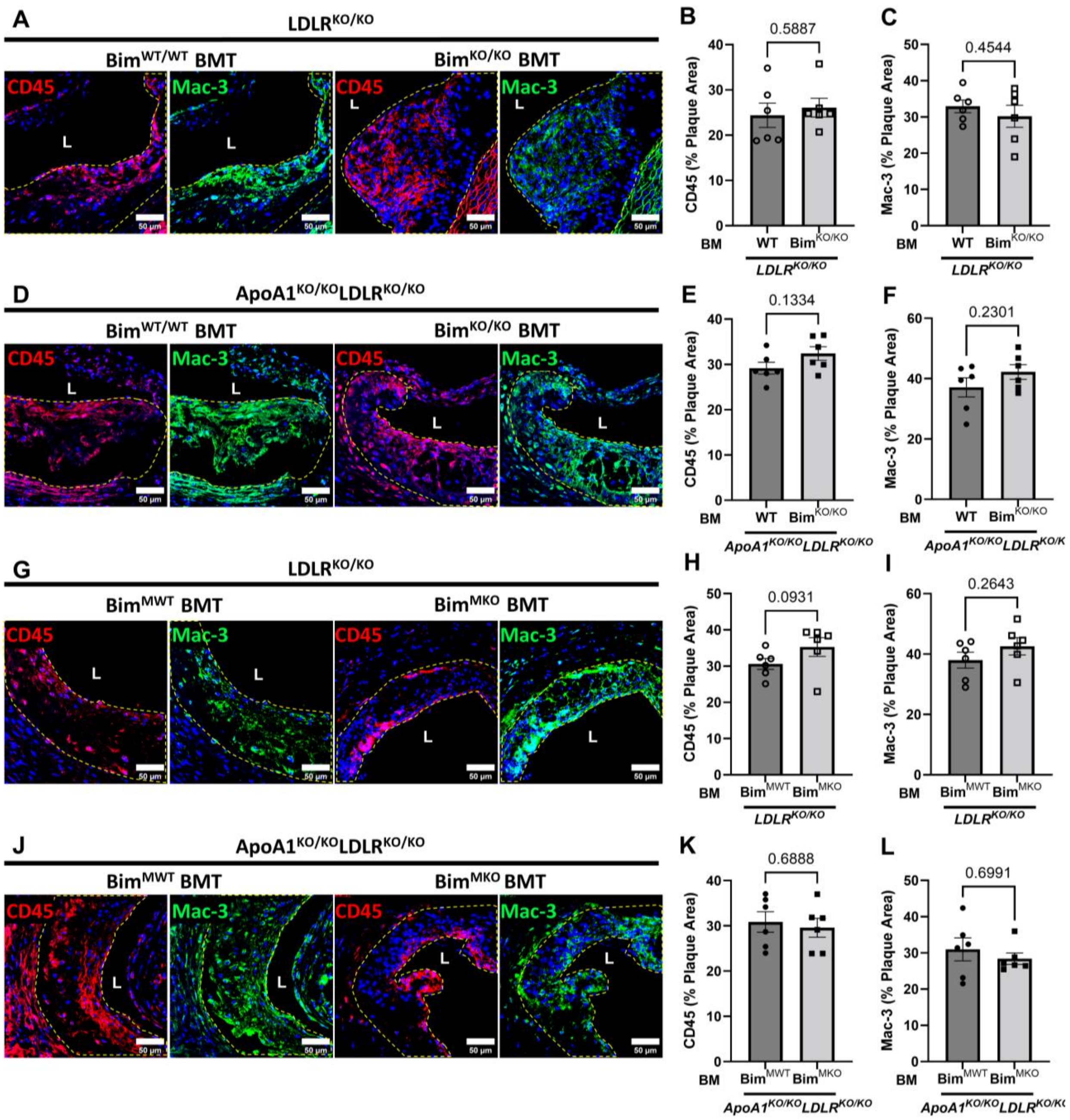

Supplemental Figure 21. CD45 and Mac-3 staining in atherosclerotic plaques from LDLR$^{KO/KO}$ and ApoA1$^{KO/KO}$LDLR$^{KO/KO}$ mice transplanted with WT, Bim$^{KO/KO}$, Bim^{MWT}, or Bim^{MKO} bone marrow fed a high-fat diet for 10 weeks.

A, Representative images and **B**, quantification of CD45 (red) and **C**, Mac-3 (green) immunofluorescent staining as a percentage of aortic sinus atherosclerotic plaque area of LDLR$^{KO/KO}$ mice transplanted with

WT or $Bim^{KO/KO}$ BM. **D**, Representative images and **E**, quantification of CD45 and **F**, Mac-3 staining as a percentage of aortic sinus atherosclerotic plaque area of $ApoA1^{KO/KO}LDLR^{KO/KO}$ mice transplanted with WT or $Bim^{KO/KO}$ BM. **G**, Representative images and **H**, quantification of CD45 and **I**, Mac-3 staining as a percentage of aortic sinus atherosclerotic plaque area of $LDLR^{KO/KO}$ mice transplanted with Bim^{MWT} or Bim^{MKO} BM. **J**, Representative images and **K**, quantification of CD45 and **L**, Mac-3 staining as a percentage of aortic sinus atherosclerotic plaque area of $ApoA1^{KO/KO}LDLR^{KO/KO}$ mice transplanted with Bim^{MWT} or Bim^{MKO} BM. Yellow dashed line outlines the plaque area. "L" represents the lumen of the aortic valve leaflet. For **B and H**, statistical analysis was done using Mann Whitney test. For **C – F**, and **I – L**, statistical analysis was conducted using unpaired t test. Statistical significance is considered when $p<0.05$. Data represents mean ± SEM.
